# Machine learning-based identification of targeted metabolomic biomarkers for early diagnosis and fibrosis-stage discrimination in metabolic dysfunction-associated steatotic liver disease

**DOI:** 10.3389/fnut.2026.1849329

**Published:** 2026-08-24

**Authors:** Shimaa Abdelsattar, Hiba S. Al-Amodi, Hala F. M. Kamel, Mahmoud Nazih, Arwa Fadel Flemban, Sabry M. Abdelmegeed, Naglaa Abdelmawgoud Ahmed, Ehab Darwish, Ayman Ahmed Sakr, Yousef M. Abdelgawad, Basma M. Abdelgawad, Inas Moaz, Elaf Abozeid, Hanan M. Bedair, Mai Abozeid, Mervat Abdelkreem, Shimaa K. Zween

**Affiliations:** 1 Clinical Biochemistry and Molecular Diagnostics Department, National Liver Institute, Menoufia University, Shebin El-Kom, Egypt; 2 Biochemistry Department, Faculty of Medicine, Umm Al-Qura University, Makkah, Saudi Arabia; 3 Medical Biochemistry and Molecular Biology Department, Faculty of Medicine, Ain Shams University, Cairo, Egypt; 4 Clinical Pharmacy Department, Faculty of Pharmacy, Ahram Canadian University (ACU), Giza, Egypt; 5 Scientific Office, Egyptian Society of Pharmacogenomics and Personalized Medicine (ESPM), Cairo, Egypt; 6 Clinical Pharmacy Department, Faculty of Pharmacy, Ahram Canadian University (ACU), October 6 City, Giza, Egypt; 7 Pathology Department, Faculty of Medicine, Umm Al-Qura University, Makkah, Saudi Arabia; 8 Community Health Nursing Department, Faculty of Nursing, Menoufia University, Shebin El-Kom, Egypt; 9 Faculty of Nursing, Al Ryada University for Science and Technology, Sadat City, Egypt; 10 Hepatology, Gastroenterology and Infectious Diseases Department, Faculty of Medicine, Zagazig University, Zagazig, Egypt; 11 Tropical Medicine Department, Faculty of Medicine, Menoufia University, Shebin El-Kom, Egypt; 12 Faculty of Medicine, Menoufia University, Shebin El-Kom, Egypt; 13 Faculty of Medicine, Menoufia National University, Menoufia, Egypt; 14 Epidemiology and Preventive Medicine Department, National Liver Institute, Menoufia University, Shebin El-Kom, Egypt; 15 Clinical Pathology Department, National Liver Institute, Menoufia University, Shebin El-Kom, Egypt; 16 Hepatology and Gastroenterology Department, National Liver Institute, Menoufia University, Shebin El-Kom, Egypt; 17 Internal Medicine Department, Faculty of Medicine, Menoufia University, Shebin El-Kom, Egypt

**Keywords:** acylcarnitines, amino acids, fibrosis-stage discrimination, machine-learning models, metabolic dysfunction-associated steatotic liver disease, metabolomics, urinary organic acids

## Abstract

**Background:**

Metabolically dysfunction-associated steatotic liver disease (MASLD) is the most prevalent liver disease worldwide and is increasing in parallel with metabolic syndrome and obesity. In this exploratory, cross-sectional study, we analysed metabolic changes in the blood and urine of patients with early-stage MASLD (fibrosis grades 0, 1, and 2) to identify metabolites associated with disease presence and the prevalent fibrosis stage and to develop machine learning models for case discrimination and fibrosis-stage stratification.

**Methods:**

Fifty-one metabolites, including17 urinary organic acids, 14 blood amino acids, 19 blood acylcarnitines/free carnitine, and glucose, were quantified in 232 participants (100 controls and 132 patients with MASLD: 68 F0, 34 F1, and 30 F2) using gas chromatography–mass spectrometry (GC/MS) and tandem mass spectrometry (MS/MS). MASLD-associated metabolites were visualised using volcano plots, cluster heatmaps, and a metabolic network diagram. Three machine learning approaches, namely, orthogonal partial least squares discriminant analysis (OPLS-DA), random forest (RF), and support vector machines (SVM), were implemented within a strictly leakage-free pipeline (feature selection and preprocessing performed within training folds only), with performance evaluated on independent test sets and validated by permutation testing and repeated cross-validation.

**Results:**

A case-discrimination panel *comprised β*-hydroxy butyrate, adipic acid, acylcarnitines (C0, C2, C3, C10:1, C14:1, and C18:1), formiminoglutamate, glucose, glycine, citric and lactic acids, Leu/Ile, pyroglutamate, sebacic and suberic acids, tiglylglycine, and valine. A stage-stratification panel comprising valine, ethylmalonate, glycine, acylcarnitines (C0, C8:1, C2, C5, C16, C10, C18, and C18:1), Leu/Ile, alanine, glutamine, pyroglutamate, 3-hydroxybutyrate, succinate, vanillylmandelate, and citrulline was associated with MASLD severity. Metabolite-based models discriminated disease status more effectively than FIB-4 (AUC 0.74) and APRI (AUC 0.70) in this cohort; permutation testing (OPLS-DA permutation *p* ≤ 0.001; random-forest empirical p ≈ 0.01) confirmed the models captured genuine biological structure rather than random patterns.

**Conclusion:**

Machine learning applied to targeted blood and urinary metabolomics identified candidate metabolite signatures associated with early-stage MASLD and fibrosis stage. These findings are exploratory and hypothesis-generating; prospective, externally validated studies with metabolically matched comparators are required before clinical application.

## 1 Introduction

Metabolically dysregulated steatotic liver disease (MASLD) encompasses a broad spectrum of liver disease, ranging from simple steatosis to the more severe metabolic dysfunction-associated steatohepatitis (MASH) (1). MASLD is highly prevalent, with a worldwide occurrence approaching 30%, and its prevalence is increasing in parallel with obesity and metabolic syndrome (MetS) (2, 3). It is associated with progressive liver fibrosis and its sequelae of portal hypertension and end-stage liver disease, and ultimately hepatocellular carcinoma (4), and is increasingly recognized as a global public health concern (5) with extrahepatic manifestations including cancer (6) and cardiovascular disease (7).

In 2023, a global consensus replaced the terms “non-alcoholic fatty liver disease” (NAFLD) and “non-alcoholic steatohepatitis” (NASH) with “MASLD” and “MASH,” respectively, and established new diagnostic criteria based on the presence of steatosis together with at least one of five cardiometabolic risk factors (obesity, elevated triglycerides, low HDL cholesterol, dysglycaemia, and hypertension) (8). Subsequent studies have demonstrated near-complete overlap between groups classified by MASLD and NAFLD (9–12).

MASLD is regarded as the hepatic manifestation of MetS, and its pathogenesis is linked to insulin resistance (IR) and altered hepatic metabolism; it is a significant risk factor for type 2 diabetes mellitus (T2DM) (13, 14). It is characterised by hepatic fat content ≥ 5% on imaging or histology, in the absence of significant alcohol consumption and other secondary causes. Among individuals with MASLD, liver fibrosis is the principal determinant of both overall mortality and liver-related events. Ultrasound remains the recommended initial imaging method for moderate-to-severe steatosis and is widely used for screening (15). Histological assessment is the reference standard for staging fibrosis, but biopsy is poorly suited to a disorder of this frequency because of its cost and associated risks. Vibration-controlled transient elastography (TE) is a quick, painless, and well-tolerated measure of liver stiffness, currently recommended as a clinically useful and reasonably accurate method for detecting significant fibrosis (≥ F2) (16). Nonetheless, there is no reliable non-invasive method for detecting MASLD in its early stages (F0–F2); liver enzyme levels in these patients may be normal (17), and studies of risk determinants and prediction scores have produced inconsistent findings (18–21).

Identifying patients at risk is therefore crucial for the rational allocation of limited resources, particularly as several liver-specific therapies are approaching clinical use. Reliable and accessible tools are also needed to monitor disease over time, evaluate treatment, and act as companion diagnostics (7). Because the liver produces energy through numerous metabolic pathways, hepatic dysfunction alters intermediate metabolites; MASLD is also associated with adipocyte dysfunction, genetic factors, and an imbalance in hepatic energy metabolism (22). Numerous studies have investigated metabolomic markers arising from dysregulated lipid, glucose, and amino acid (AA) metabolism in MASLD (23–25), including methionine, arginine, homocysteine, branched-chain amino acids (BCAAs), and aromatic amino acids (16).

IR and lipid accumulation are thought to arise, in part, from dysfunctional *β*-oxidation of fatty acids in hepatic and muscle tissues. Fatty acids are activated to acyl-CoA and transported into mitochondria via carnitine; acylcarnitines are intermediates of this process and have emerged as indicators of aberrant fatty acid oxidation (FAO) that may serve as screening markers for MASLD. Serum acylcarnitine profiles change markedly with MASLD progression (26), although their relationship with disease status remains incompletely understood.

Urine has emerged as a valuable source of non-invasive biomarkers (27). Urinary metabolites are less affected by sampling time and diurnal variation than blood-based indicators, are easy to collect, exhibit little biological variance, and provide insight into ongoing metabolic processes (28). Urinary organic acid testing has long been used to diagnose metabolic diseases and inborn errors of metabolism (29, 30) and can reveal intermediates associated with hepatic steatosis or fibrosis (31). Machine learning tools can build predictive models from data without explicit programming, and its application in gastroenterology is expanding (32); compared with traditional statistics, they can efficiently analyse large, high-dimensional datasets and identify the most discriminative markers for risk stratification (33–35).

Although several metabolomic studies have examined blood-based (36) or urinary (31, 37) MASLD biomarkers in isolation, studies combining targeted metabolomics from both matrices are rare. Blood reflects systemic metabolic status, and urine reflects renal excretion of metabolic end-products; together, they offer a complementary, two-dimensional window into MASLD pathophysiology. To our knowledge, this is among the first studies to apply a dual-matrix targeted metabolomic approach (blood acylcarnitines and amino acids by MS/MS; urinary organic acids by GC/MS) combined with three machine learning algorithms for stage-specific MASLD biomarker discovery.

Accordingly, this exploratory study examined metabolic changes in individuals with early-stage MASLD. It identifies metabolites associated with disease presence and fibrosis stages (F0, F1, and F2) to develop machine learning models for case discrimination and fibrosis-stage stratification.

## 2 Patients and methods

This exploratory, cross-sectional case–control study included 132 patients with MASLD recruited from the Endocrinology Outpatient Clinic of the National Liver Institute and the Internal Medicine Department of the Faculty of Medicine, Menoufia University, Egypt. Patients had sonographic signs of steatosis (bright, enlarged liver). FIB-4 and FibroScan were used exclusively for fibrosis staging, not for case ascertainment, to avoid selection bias towards older patients or those with elevated transaminases, which would undermine the study’s focus on early-stage detection.

Patients were excluded if they had: (i) secondary causes of hepatic steatosis, including alcohol consumption, hepatitis B or C, autoimmune hepatitis, primary biliary cholangitis, primary sclerosing cholangitis, drug-induced liver injury, or Wilson disease; (ii) advanced liver disease, including established cirrhosis, hepatocellular carcinoma, or portal hypertension of any aetiology; (iii) significant comorbidity that independently alters metabolite profiles, including chronic kidney disease (eGFR < 30 mL/min/1.73 m^2^) or ischaemic heart disease; and (iv) treatment with medications known to cause hepatic steatosis, including corticosteroids, amiodarone, tamoxifen, and valproate.

A control group of 100 apparently healthy blood donors of similar age and sex was included. Controls underwent abdominal ultrasonography with a normal hepatic appearance and were excluded if any sonographic steatosis was present; they also had normal liver function test results and negative hepatitis B/C and autoimmune serology. All participants provided written informed consent. The study was approved by the Faculty of Medicine and the Menoufia University Ethics Committee (IRB approval 6/2025 INT).

*Participant flow (STROBE)*. Consecutive patients with sonographic steatosis were screened; individuals meeting any exclusion criterion (competing aetiology, advanced disease, confounding comorbidity, or steatogenic medication) were excluded, and controls with any sonographic steatosis, abnormal liver function, or positive serology were also excluded. The final analysed cohort comprised 232 participants (100 controls and 132 patients with MASLD: 68 F0, 34 F1, and 30 F2).

Every participant underwent a full medical history and physical examination, including anthropometric measurements (height, weight, waist circumference [WC] at the top of the iliac crests, and hip circumference [HC] at the widest point of the buttocks, measured with a non-stretchable tape; weight to the nearest 0.1 kg on a calibrated digital scale without shoes). Body mass index (BMI) was calculated as weight (kg)/height^2^ (m^2^). The Visceral Adiposity Index was calculated using sex-specific formulae, and the Lipid Accumulation Product was calculated as [(WC − 65) × TG] in men and [(WC − 58) × TG] in women (TG in mmol/L) (38). Insulin resistance was estimated using the Homeostatic Model Assessment for Insulin Resistance (HOMA-IR) [fasting insulin (μU/mL) × fasting glucose (mg/dL)/405] (39).

### 2.1 Assessment of liver fibrosis

Liver stiffness measurement (LSM) was performed using vibration-controlled transient elastography (FibroScan, Echosens, France) in the right intercostal space, with the participant in the supine position and the right arm fully extended, during breath-hold. Fibrosis was staged, not diagnosed by biopsy, using validated LSM cut-offs (16, 40–42):

- F0 (no fibrosis): LSM < 5.5 kPa.
- F1 (mild fibrosis): LSM 5.5–6.9 kPa.
- F2 (moderate fibrosis): LSM 7.0–8.7 kPa.
- F3–F4 (advanced fibrosis/cirrhosis; excluded from this study): LSM ≥ 8.8 kPa.

A valid LSM required ≥ 10 successful measurements, an interquartile range (IQR)/median ≤ 30%, and a success rate ≥ 60% (standard Echosens criteria). FIB-4 and APRI were computed as independent corroborating fibrosis indices (Table 1) (16, 41, 43). APRI was calculated as (AST/upper limit of normal AST) × 100/platelet count (10^9^/L); FIB-4 = (AST × age)/(platelet count [10^9^/L] × √ALT).

**Table 1:** Demographic, anthropometric, and laboratory characteristics of the study cohort (*n* = 232; 100 controls and 132 patients with MASLD).

Variable	F0 (*n* = 68)	F1 (*n* = 34)	F2 (*n* = 30)	Controls (*n* = 100)	*p*-value	*Post-hoc*
Age (years)	40.0 (33.0–46.5)	43.0 (38.0–46.5)	42.5 (40.0–45.0)	40.3 (32.0–46.5)	0.05	NA
Female, *n* (%)	34 (50.0)	16 (47.1)	16 (53.3)	48 (48.0)	0.95	NA
BMI (kg/m^2^)	30.0 (27.1–33.0)	31.0 (29.6–33.8)	32.0 (30.2–34.0)	21.7 (20.6–22.4)	<0.001	a,b,c
Waist circumference (cm)	103.0 (97.9–110.9)	101.0 (95.2–110.1)	106.0 (98.9–111.5)	69.0 (66.2–71.9)	<0.001	a,b,c
Hip circumference (cm)	112.0 (108.5–116.2)	112.0 (100.7–122.0)	119.5 (108.0–129.4)	89.5 (84.8–94.4)	<0.001	a,b,c
Waist-to-hip ratio	0.93 (0.88–0.98)	0.91 (0.86–1.00)	0.91 (0.83–0.98)	0.77 (0.74–0.80)	<0.001	a,b,c
Systolic BP (mmHg)	120 (110–135)	120 (110–140)	130 (110–140)	120 (100–125)	<0.001	a,b,c,d,e
Diastolic BP (mmHg)	80 (80–80)	80 (80–85)	80 (80–90)	80 (70–80)	<0.001	a,b,c,e
Glucose (mg/dL)	90.0 (76.0–99.0)	90.0 (79.0–97.0)	91.0 (80.0–97.0)	82.0 (73.0–91.0)	<0.001	a,b,c
HbA1c (%)	4.70 (4.10–5.30)	4.90 (4.50–5.30)	5.32 (4.52–6.12)	4.65 (4.15–5.15)	<0.001	a,b,c,d,e
Insulin (μU/mL)	35.0 (20.4–49.6)	30.0 (14.3–45.7)	39.5 (25.5–53.5)	10.3 (7.4–13.2)	<0.001	a,b,c
ALT (U/L)	42.0 (23.0–61.0)	58.0 (36.0–80.0)	41.0 (15.0–67.0)	16.0 (9.0–23.0)	<0.001	a,b,c
AST (U/L)	31.0 (16.0–46.0)	33.0 (9.0–57.0)	36.5 (11.5–61.5)	9.0 (6.0–12.0)	<0.001	a,b,c
HDL-C (mg/dL)	62.0 (24.0–100.0)	60.0 (36.5–83.5)	54.5 (31.5–77.5)	63.0 (55.3–70.8)	<0.001	a,b,c
Triglycerides (mg/dL)	120.0 (40.5–199.5)	150.0 (75.5–224.5)	170.0 (124.0–216.0)	101.0 (82.5–119.5)	<0.001	a,b,c
Total cholesterol (mg/dL)	242.0 (158.0–326.0)	246.0 (112.0–380.0)	249.5 (215.5–283.5)	148.5 (132.8–164.3)	<0.001	a,b,c
Haemoglobin (g/dL)	13.2 (11.1–15.3)	13.0 (11.4–14.6)	12.5 (11.5–13.5)	12.5 (11.5–13.5)	0.001	a,b
Creatinine (mg/dL)	0.60 (0.20–1.00)	0.70 (0.55–0.85)	0.70 (0.60–0.80)	0.70 (0.60–0.80)	0.22	NA
Albumin (g/dL)	4.80 (4.30–5.30)	4.50 (3.85–5.15)	4.40 (3.70–5.10)	4.60 (3.80–5.40)	<0.001	a,b,d
Total bilirubin (mg/dL)	0.60 (0.20–1.00)	0.60 (0.40–0.80)	0.60 (0.40–0.80)	0.20 (0.10–0.30)	<0.001	a,b,c
Alkaline phosphatase (U/L)	89.0 (70.0–108.0)	98.0 (80.5–115.5)	93.0 (83.0–103.0)	80.0 (61.5–98.5)	<0.001	a,b,c
FIB-4 index	0.52 (0.36–0.68)	0.82 (0.47–1.17)	0.85 (0.59–1.11)	0.20 (0.10–0.30)	<0.001	a,b,c,e
APRI	0.25 (0.13–0.37)	0.34 (0.10–0.58)	0.36 (0.20–0.52)	0.06 (0.04–0.08)	<0.001	a,b,c,e
Portal vein diameter (mm)	11.0 (10.0–12.0)	11.0 (10.0–12.0)	12.0 (11.0–13.0)	9.0 (7.0–11.0)	<0.001	a,b,c,d,e

Values are median (interquartile range). Comparisons by Kruskal–Wallis test with Dunn’s post-hoc correction. *Post-hoc*: a = F0 vs. control; b = F1 vs. control; c = F2 vs. control; d = F0 vs. F1; e = F0 vs. F2. BMI, body mass index; ALT, alanine aminotransferase; AST, aspartate aminotransferase; HDL-C, high-density lipoprotein cholesterol; APRI, AST-to-platelet ratio index; FIB-4, Fibrosis-4 index. Full ranges are given in Supplementary Table S1.

MASLD was defined according the 2023 multi-society consensus (8): (1) hepatic steatosis on imaging or histology; (2) at least one of five cardiometabolic criteria (BMI ≥ 25 kg/m^2^, or ≥ 23 in Asians; fasting glucose ≥ 5.6 mmol/L, T2DM, or antidiabetic therapy; blood pressure ≥ 130/85 mmHg or antihypertensive therapy; triglycerides ≥ 1.70 mmol/L or lipid-lowering therapy; HDL-C < 1.0 mmol/L in men or < 1.3 mmol/L in women); and (3) exclusion of competing causes of liver disease.

### 2.2 Sample collection and laboratory investigations

Five millilitres of venous blood were collected from all participants after a standardised 8-h overnight fast. The sample was divided into 3 mL in a plain tube (for serum biochemistry and the insulin ELISA) and 2 mL in an EDTA tube (for plasma HbA1c; Sysmex XT-1800i, Japan). Lipid profile (LDL-C, HDL-C, total cholesterol, and triglycerides), 2-h post-prandial glucose, fasting glucose, and creatinine were measured using a Beckman Coulter Synchron CX9-ALX analyser (Beckman Instruments, Fullerton, CA, USA). Dried blood spots from a sterile thumb puncture were collected on Guthrie cards (GE Healthcare, NJ, USA), air-dried, and stored at −80 °C. Midstream urine was collected in sterile, preservative-free containers, placed on ice, centrifuged at 2,000 × g for 10 min at 4 °C, and aliquoted within 2 h for creatinine and albumin (albumin-to-creatinine ratio) on the same analyser (44); aliquots for organic-acid analysis were stored at −80 °C until batch processing by GC/MS.

### 2.3 Serum fasting insulin

Serum insulin was measured using a double-antibody sandwich ELISA (Cat. 201–12-1720; Shanghai Sunred Biological Technology Co., Ltd).

### 2.4 Chemicals and reagents

MaChrom® amino acid and acylcarnitine kits for dried-blood-spot (non-derivatised) analysis were obtained from Chromsystems Instruments & Chemicals GmbH (Munich, Germany). Pentadecanoic acid (PDA; Acros Organics, NJ, USA) was used as the internal standard (IS). N, O-bis(trimethylsilyl)trifluoroacetamide (BSTFA) with 1% trimethylchlorosilane (TMCS) (Supelco, Bellefonte, PA, USA) was used as the derivatising reagent; HPLC-grade methanol (Fisher Scientific, Loughborough, UK) was used as the solvent.

### 2.5 Acylcarnitine and amino acid assays by tandem mass spectrometry (MS/MS)

Free carnitine (C0), acetyl- (C2), propionyl- (C3), butyryl- (C4), isovaleryl- (C5), hydroxyisovaleryl- (C5-OH), glutaryl- (C5-DC), hexanoyl- (C6), octanoyl- (C8), decanoyl- (C10), dodecanoyl- (C12), myristoyl- (C14), tetradecenoyl- (C14:1), palmitoyl- (C16), stearoyl- (C18), and oleoyl-carnitine (C18:1) were quantified by MS/MS following a published method (45) with minor modifications. In brief, a 3-mm dried-blood-spot punch was extracted with lyophilised IS in extraction buffer, shaken at 600 rpm for 25 min, and a 10 μL aliquot was injected into an Acquity UPLC H-Class MS/MS system (Waters, MA, USA) operating in multiple reaction monitoring mode. Quantification was performed using isotope-labelled internal standards and expressed as μmol/L (NeoLynx software) by comparing analyte and IS signal intensities.

### 2.6 Quantitative urinary organic-acid assay by GC/MS

Frozen urine samples were incubated at 37 °C for 14 min and vortexed; each metabolite was normalised to urinary creatinine (mmol/mol creatinine) to control for urine concentration (46). Extraction and derivatisation followed a published method (41) with minor changes; the PDA stock IS was prepared by dissolving 50 mg in 100 mL of methanol. One microlitre of the derivatised sample was injected in split mode into an Agilent 7,890 GC (30.0 m × 0.25 mm i.d. fused-silica capillary column, 0.25 μm HP-5 MS phase; Agilent, USA), injector 250 °C, helium 1 mL/min. The oven temperature was maintained at 80 °C for 2 min, increased at 4 °C/min to 280 °C, and the held for 3 min; the MS quadrupole temperature was maintained at 150 °C and the ion source temperature at 230 °C. Seven-point calibration curves (m/z 50–550) were constructed by linear regression of analyte-to-IS peak-area ratios in selective-ion-monitoring mode. The results were expressed as μmol/mmol creatinine (GC ChemStation, Agilent).

### 2.7 Quality control, data analysis, and machine learning models

*Quality control*. Quantification relied on isotope-labelled internal standards and seven-point calibration curves; urinary analytes were normalised to creatinine to control for concentration. A pre-specified missingness threshold was applied, and missing values were imputed by K-nearest neighbours (KNN) within the training set only. Analytes that failed the coefficient of variation criteria were excluded, values below the limit of detection were handled using a fixed substitution rule, and samples were processed batch-wise with internal standard-based correction.

Fifty-one analytes (19 blood carnitines/acylcarnitines, 14 blood amino acids, 17 urinary organic acids, and glucose) were measured in 232 participants (100 controls; 132 patients: 68 F0, 34 F1, and 30 F2). Raw concentrations were preprocessed using median normalisation, log transformation, and scaling (mean centring and division by the standard deviation). Exploratory analysis included hierarchical clustering of metabolite classes, visualised as heatmaps (ggplot2, R v4.3.2). Comparative analysis were performed using OPLS-DA, RF, and SVM (ropls, randomForest, e1071, and ggplot2 packages).

*Multiplicity and univariate testing*. Between-group differences in metabolite levels were assessed using Student’s *t*-test in MetaboAnalyst v6.0; differentially abundant metabolites were defined by a fold-change threshold of |1.5| and a Benjamini-Hochberg false discovery rate (FDR)-adjusted *p*-value of < 0.05. Group medians with IQRs, along with both raw and FDR-adjusted *p*-values, are provided in Supplementary Tables S2, S3.

*Sensitivity analysis for metabolic confounding*. To assess whether metabolite differences were driven primarily by obesity and insulin resistance rather than MASLD-specific pathology, multivariate logistic regression was performed, adjusting for BMI, HOMA-IR, and triglycerides. For each pairwise comparison, the top 20 discriminant metabolites were entered; those remaining significant after adjustment (Benjamini-Hochberg-corrected *p* < 0.05) were considered the most robust candidates, with adjusted odds ratios and 95% confidence intervals reported in Supplementary Table S3.

For OPLS-DA, performance metrics included accuracy, Q^2^ (predictive ability), and R^2^ (explained variance), assessed by 10-fold cross-validation with three components to minimise overfitting; model significance was confirmed by permutation testing (1,000 permutations; permutation p for R^2^Y and Q^2^ = 0.001–0.01). RF used 1,000 trees with the number of variables per split optimised as the square root of the total. SVM performance was evaluated based on the magnitude of the model coefficients. Convergent criteria selected key metabolites: fold change ≥ 1.5 with adjusted *p* < 0.05, and the top-ranked features by VIP score (OPLS-DA), mean decrease in accuracy (RF), and SVM coefficients. Pathway enrichment analysis was performed using MetaboAnalyst v6.0 (hypergeometric test; adjusted p < 0.05).

*Operational definitions*. As this study is cross-sectional, the term “diagnostic” is used strictly to denote the ability to distinguish groups on a single occasion based on metabolite profiles, and “stage stratification” (previously “prognostic”) denotes discrimination of prevalent fibrosis severity in established MASLD. These terms do not imply prediction of future clinical outcomes; prospective validation in independent cohorts is required before clinical implementation.

#### 2.7.1 Machine learning workflow and leakage-free validation framework

To ensure robust, leakage-free evaluation, the following pipeline was applied (see also Supplementary Figure S16).

*Data splitting*. For RF and SVM, the dataset was divided into training (70%) and independent test (30%) subsets by stratified random sampling to preserve class balance. For OPLS-DA, 10-fold cross-validation was applied within the training set. Each pairwise comparison used its own independent stratified split of the two relevant groups. As the 100 controls are shared across the three case-detection comparisons, controls were repartitioned per comparison, and preprocessing and feature selection were re-derived from scratch within each comparison’s training set.

*Preprocessing*. All preprocessing steps (KNN imputation, median normalisation, log-transformation, mean-centring, and scaling) were performed exclusively on the training set; the learned parameters were then applied to the test set. No test data were used in any preprocessing step.

*Feature selection*. Variable importance (VIP for OPLS-DA; mean decrease in accuracy for RF; SVM coefficients) was computed within the training folds only; no test observation influenced feature ranking.

*Hyperparameter tuning*. For RF, the number of variables sampled per split (mtry) was optimised by minimising out-of-bag error across mtry = 2, 4, 7, 10, and 14 within the training set, with ntree fixed at 1,000 after the OOB error plateaued. SVM cost (C ∈ {0.01, 0.1, 1, 10, 100}) and kernel width (gamma ∈ {0.001, 0.01, 0.1, 1}) were optimised by 5-fold cross-validated grid search within the training set, using a radial-basis-function kernel.

*Cross-validation and evaluation*. OPLS-DA used 10-fold cross-validation, whereas RF and SVM used 5-fold stratified cross-validation within the training set for hyperparameter optimisation, with final performance evaluated on the held-out test set. Performance metrics included accuracy (with 95% binomial confidence intervals), sensitivity, specificity, positive and negative predictive values, F1 score, area under the ROC curve (with 95% bootstrap confidence intervals), and calibration (Hosmer–Lemeshow test). Performance is reported in Table 2 and Supplementary Table S4. As an additional, fully leakage-free robustness analysis, the complete pipeline (preprocessing, feature selection, hyperparameter tuning and evaluation) was repeated using repeated nested cross-validation (5 outer folds × 10 repeats = 50 held-out evaluations, with inner 5-fold tuning) and Boruta all-relevant feature selection; this analysis reproduced the main findings, including the same, more demanding adjacent-stage F1-vs-F0 boundary, and is reported in Supplementary Tables S4b,c.

**Table 2:** Classification performance of the machine learning models for MASLD case discrimination and fibrosis-stage stratification, with 95% confidence intervals.

Comparison	Model	Accuracy (95% CI)	Sens.	Spec.	AUC (95% CI)	Notes
Controls vs. F0	OPLS-DA	1.00 (0.93–1.00)	1.00	1.00	1.00 (0.93–1.00)	Test *n* = 50; perm. pQ^2^ = 0.01
	SVM	1.00 (0.93–1.00)	1.00	1.00	1.00 (0.93–1.00)	Test set; 10-fold CV
	RF	1.00 (0.93–1.00)	1.00	1.00	1.00 (0.93–1.00)	Test set; 1,000 trees
Controls vs. F1	OPLS-DA	1.00 (0.91–1.00)	1.00	1.00	1.00 (0.91–1.00)	Test *n* = 40; perm. pQ^2^ = 0.01
	SVM	1.00 (0.91–1.00)	1.00	1.00	1.00 (0.91–1.00)	Test set; 10-fold CV
	RF	1.00 (0.91–1.00)	1.00	1.00	1.00 (0.91–1.00)	Test set; 1,000 trees
Controls vs. F2	OPLS-DA	1.00 (0.91–1.00)	1.00	1.00	1.00 (0.91–1.00)	Test *n* = 39; perm. pQ^2^ = 0.01
	SVM	1.00 (0.91–1.00)	1.00	1.00	1.00 (0.91–1.00)	Test set; 10-fold CV
	RF	1.00 (0.91–1.00)	1.00	1.00	1.00 (0.91–1.00)	Test set; 1,000 trees
F0 vs. F1	OPLS-DA	1.00 (0.90–1.00)	1.00	1.00	1.00 (0.90–1.00)	Test *n* = 30; perm. pR^2^Y = 0.01
	SVM	1.00 (0.90–1.00)	1.00	1.00	1.00 (0.90–1.00)	Test set; 10-fold CV
	RF	1.00 (0.90–1.00)	1.00	1.00	1.00 (0.90–1.00)	Test set; 1,000 trees
F0 vs. F2	OPLS-DA	1.00 (0.91–1.00)	1.00	1.00	1.00 (0.91–1.00)	Test *n* = 29; perm. pR^2^Y = 0.01
	SVM	1.00 (0.91–1.00)	1.00	1.00	1.00 (0.91–1.00)	Test set; 10-fold CV
	RF	1.00 (0.91–1.00)	1.00	1.00	1.00 (0.91–1.00)	Test set; 1,000 trees

AUC, area under the ROC curve; CI, confidence interval; CV, cross-validation; Sens., sensitivity; Spec., specificity. All metrics were derived from independent test sets (30% of the data) not used during training; OPLS-DA additionally used 10-fold cross-validation and permutation testing (*p* ≤ 0.001 across 1,000 permutations). With small test sets (*n* = 29–50), a point estimate of 1.00 carries a wide confidence interval and denotes excellent discrimination with wide uncertainty rather than literal perfection; calibration was adequate for all models (Hosmer–Lemeshow *p* > 0.05, Supplementary Table S4). By comparison, FIB-4 and APRI achieved AUCs of 0.74 and 0.70 in this cohort.

*Code and data availability*. The complete R analysis code (preprocessing, imputation, scaling, feature selection, model training, hyperparameter tuning, cross-validation, permutation testing, and test-set evaluation) and the processed, de-identified metabolite matrix will be deposited in a public repository (GitHub with a Zenodo DOI; metabolomic data in MetaboLights) and will also be provided as Supplementary materials, so that all results can be independently reproduced and the leakage-free workflow can be inspected.

## 3 Results

Participants with MASLD exhibited notable differences in anthropometric characteristics compared with controls (Table 1). Median BMI, waist circumference, and waist-to-hip ratio increased progressively from controls to F2, with the highest medians in F2 (BMI 32.6; waist circumference 112 cm); all differences were statistically significant (*p* < 0.001), whereas age and sex did not differ significantly between the groups (*p* = 0.05 and *p* = 0.95, respectively).

Routine laboratory and liver function markers also varied significantly (Table 1). ALT and AST levels were elevated in MASLD, with increasing medians across fibrosis stages (*p* < 0.001 for both). Insulin (*p* < 0.001) and FIB-4 (*p* < 0.001) also differed markedly between the groups. HDL-C declined progressively from controls to F2, while triglycerides and total cholesterol were higher in MASLD (*p* < 0.001). Significant differences were also seen in haemoglobin (*p* = 0.001) and APRI (*p* < 0.001).

Supplementary Tables S2a,b present the distribution of plasma amino acids, acylcarnitines, and urinary organic acids. Citric acid, tiglylglycine, lactic acid, tyrosine, and citrulline were higher in MASLD than in controls, with the most pronounced elevations in F2 (phenylalanine *p* = 0.001; tyrosine *p* = 0.002). Conversely, vanillylmandelate, glycine, and proline decreased as fibrosis advanced (*p* < 0.001). BCAAs (leucine, isoleucine, and valine) also differed significantly between the groups, and acylcarnitine profiles varied distinctly across MASLD stages and controls.

Supplementary Figure S1 shows a clear separation between clusters, indicating that the PLS-DA model captured differences between controls and MASLD groups across fibrosis stages (F0, F1, and F2), especially when patient characteristics and laboratory parameters were combined with metabolites as predictors. Samples closer together on the plot are more similar; points far from their group cluster may represent outliers or distinct biological states and were investigated further. Each sample has scores on the latent variables (t1 and t2) that reflect its association with the data’s patterns. Correlation structure among the measured metabolites and clinical variables is shown in Supplementary Figure S1.

### 3.1 Models to discriminate healthy individuals from MASLD patients

#### 3.1.1 Controls versus (vs.) F0

**Figure 1:**
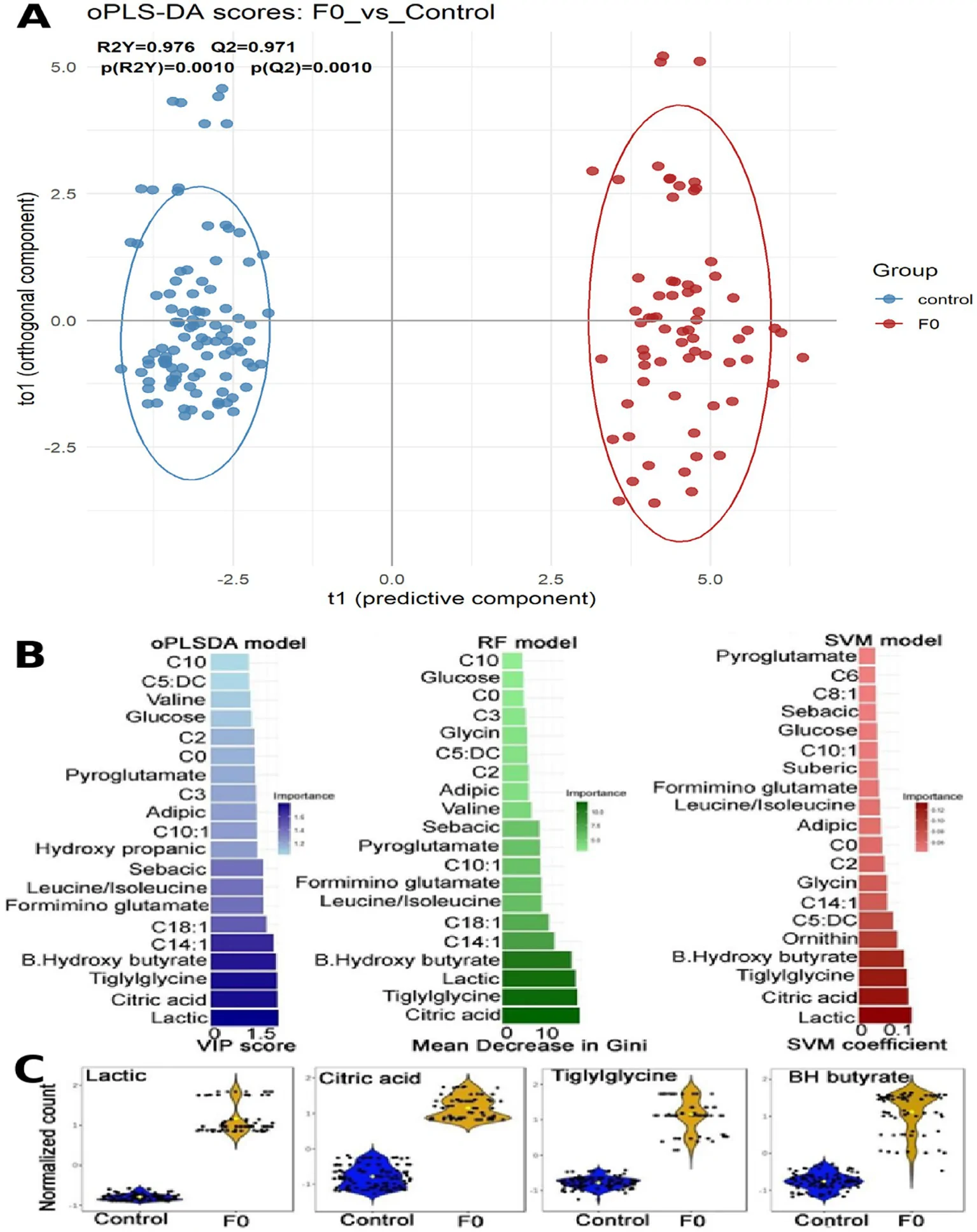
Metabolic differences between controls and F0, using OPLS-DA, RF, and SVM. **(A)** OPLS-DA score plot with 95% confidence ellipses; **(B)** Top contributing metabolites by VIP score (OPLS-DA), mean decrease in Gini (RF), and SVM coefficient; **(C)** Violin plots of representative discriminating metabolites (lactic acid, citric acid, tiglylglycine, and β-hydroxybutyrate). Lactic acid, citric acid, and tiglylglycine emerge consistently across all three models.

To examine how metabolite alterations distinguish controls from F0 (no fibrosis), we trained and validated the models on these two groups. PLS-DA gave broad separation (accuracy 0.969, R^2^ 0.664, Q^2^ 0.969), supported by OPLS-DA (R^2^X 0.353, Q^2^Y 0.975; Figure 1, Table 3). RF further validated this (accuracy 0.987); re-running RF across different training/test proportions yielded near-identical results. SVM achieved an accuracy of 1.00 (95% CI 0.93–1.00; see the honest interpretation of confidence intervals in §3.3). The intersection of RF, SVM, and OPLS-DA with univariate analyses identified the key discriminating metabolites. OPLS-DA returned 20 metabolites with VIP > 1 (Figure 1), the highest being lactic acid, citric acid, tiglylglycine, and β-hydroxybutyrate; the top RF features by mean decrease in accuracy were citric acid, tiglylglycine, lactic acid, and butyrate; and citric acid, tiglylglycine, and lactic acid dominated the SVM coefficients. Of 50 metabolites, 24 (48%) were significantly differentially abundant between controls and F0 (10 organic acids, 4 amino acids, 10 carnitines/acylcarnitines, and glucose; Supplementary Figure S2).

**Table 3:** Validation parameters for machine learning models discriminating between pairs of groups using metabolites.

Model metric	F0 vs. control	F1 vs. control	F2 vs. control	F1 vs. F0	F2 vs. F0
OPLS-DA accuracy	0.969	0.976	0.969	0.751	0.881
R^2^X	0.353	0.395	0.396	0.148	0.207
R^2^Y	0.975	0.982	0.976	0.842	0.917
Q^2^	0.969	0.976	0.969	0.751	0.881
R^2^	0.664	0.688	0.686	0.495	0.562
RMSEE	0.078	0.059	0.066	0.190	0.135
RF accuracy	0.987	1.000	1.000	0.992	0.997
SVM accuracy	1.000	0.998	1.000	1.000	1.000

OPLS-DA, orthogonal partial least squares discriminant analysis; RF, random forest; SVM, support vector machine. R^2^X, cumulative variance explained in X; R^2^Y, cumulative variance explained in Y; Q^2^, predictive ability; R^2^, coefficient of determination; RMSEE, root-mean-square error of estimation. Note the moderate, non-saturated Q^2^ (0.75 and 0.88) for the metabolically matched staging comparisons (F1 vs. F0, F2 vs. F0).

#### 3.1.2 Controls versus F1

**Figure 2:**
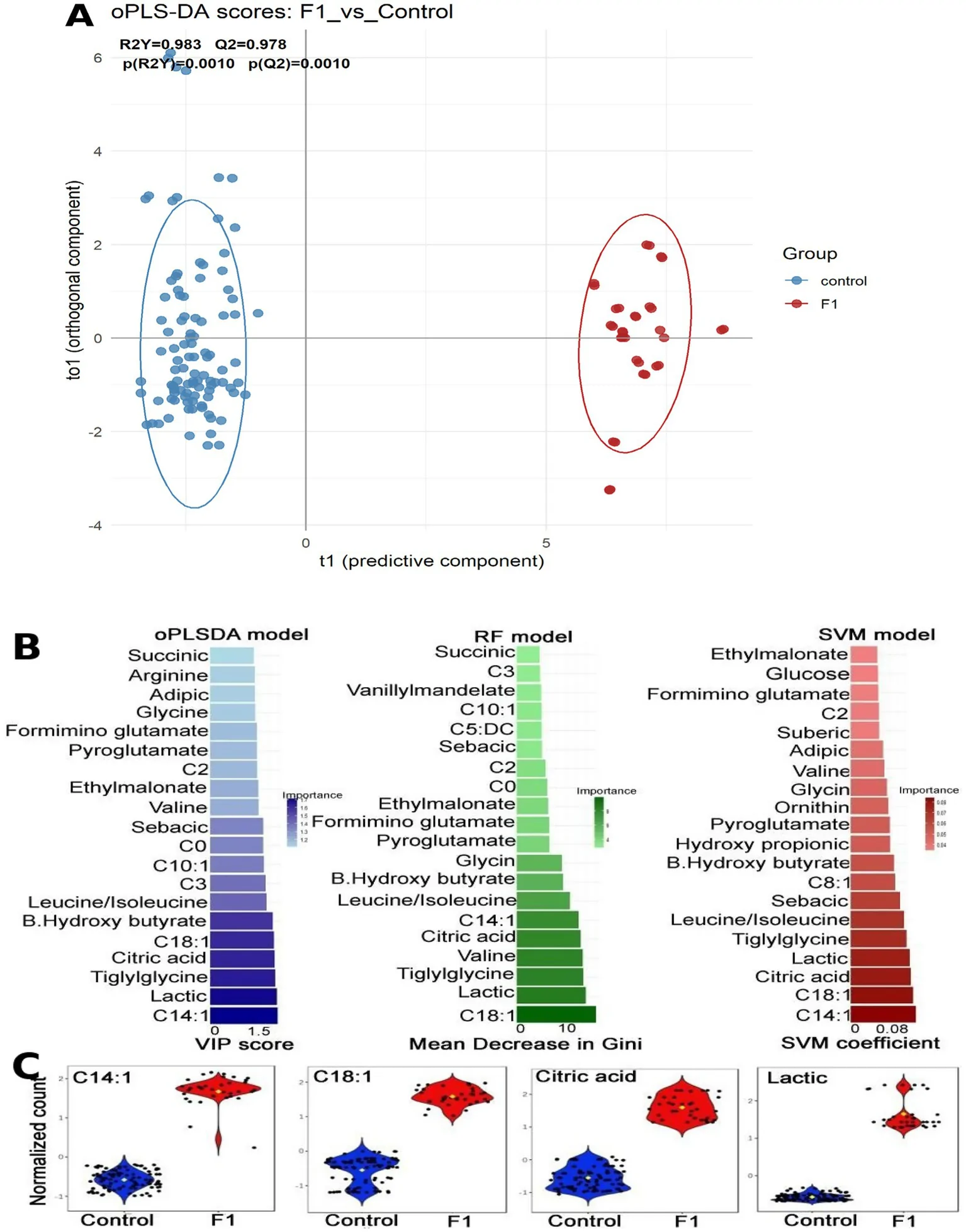
Metabolic differences between controls and F1, using OPLS-DA, RF, and SVM. Panels **(A–B)** as in Figure 1; **(C)** violin plots of representative discriminating metabolites (C14:1, C18:1, citric acid, and lactic acid).

For controls versus F1 (mild fibrosis), PLS-DA gave good separation (accuracy 0.976, R^2^ 0.688, and Q^2^ 0.976), supported by OPLS-DA (R^2^X 0.396, Q^2^Y 0.95; Figure 2, Table 3). RF and SVM corroborated this with excellent discrimination. Combining the three models with univariate analyses revealed 20 metabolites, led by C14:1, citric acid, tiglylglycine, and lactic acid; the top RF features were C18:1 (acylcarnitine), lactic acid, and tiglylglycine (organic acids) and valine (amino acid). Of 50 metabolites, 26 (52%) were differentially abundant between controls and F1 (13 organic acids, 5 amino acids, 8 carnitines/acylcarnitines, and glucose; Supplementary Figure S3).

#### 3.1.3 Controls versus F2

**Figure 3:**
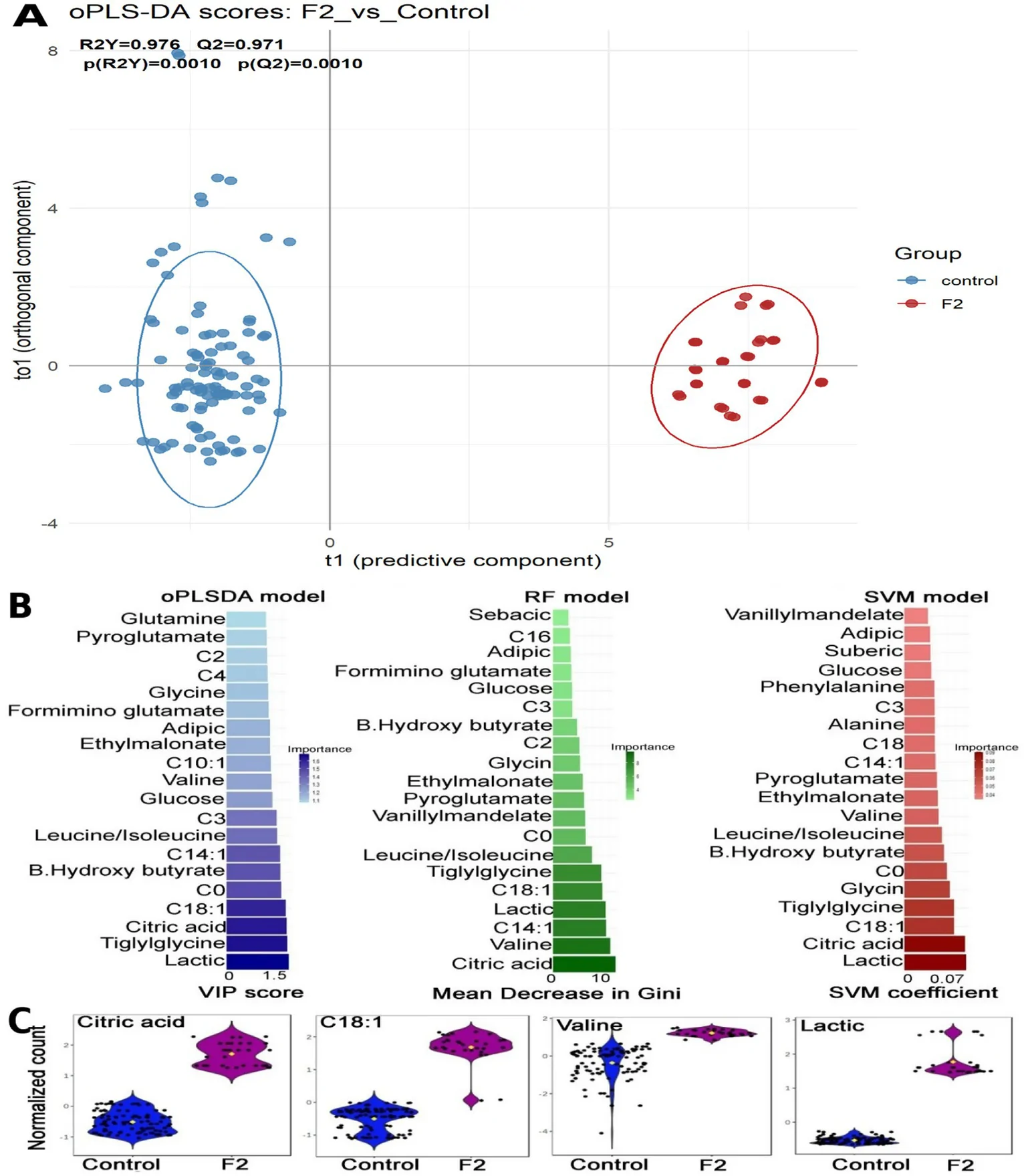
Metabolic differences between controls and F2, using OPLS-DA, RF, and SVM. Panels **(A–B)** as in Figure 1; **(C)** Violin plots of representative discriminating metabolites (citric acid, C18:1, valine, and lactic acid). Valine is displayed here as a leading random-forest discriminant (mean-decrease-in-Gini rank 2) for this comparison.

For controls versus F2 (at risk of MASH), PLS-DA showed distinct separation, supported by OPLS-DA (R^2^X 0.396, R^2^Y 0.976; Figure 3, Table 3), with RF and SVM confirming excellent discrimination. OPLS-DA returned 20 metabolites with VIP > 1, led by lactic acid, tiglylglycine, citric acid, and C18:1; the top RF features were citric acid, *β*-hydroxybutyrate, C14:1, and lactic acid; and lactic acid, citric acid, C18:1, and tiglylglycine were the leading SVM predictors. Of 50 metabolites, 26 (52%) were differentially abundant between controls and F2 (6 amino acids, 11 organic acids, 9 carnitines/acylcarnitines, and glucose; Supplementary Figure S4).

Intersecting RF, SVM, and OPLS-DA with univariate analyses produced a list of 20 metabolites that were the most important discriminators of MASLD from controls (case-discrimination panel; Panel 1). The associated metabolic pathways (Supplementary Figure S7) included glutathione metabolism, glyoxylate and dicarboxylate metabolism, neomycin/kanamycin/gentamicin biosynthesis, and valine/leucine/isoleucine biosynthesis.

### 3.2 Models for fibrosis-stage discrimination and severity stratification in established MASLD

#### 3.2.1 F0 versus F1

**Figure 4:**
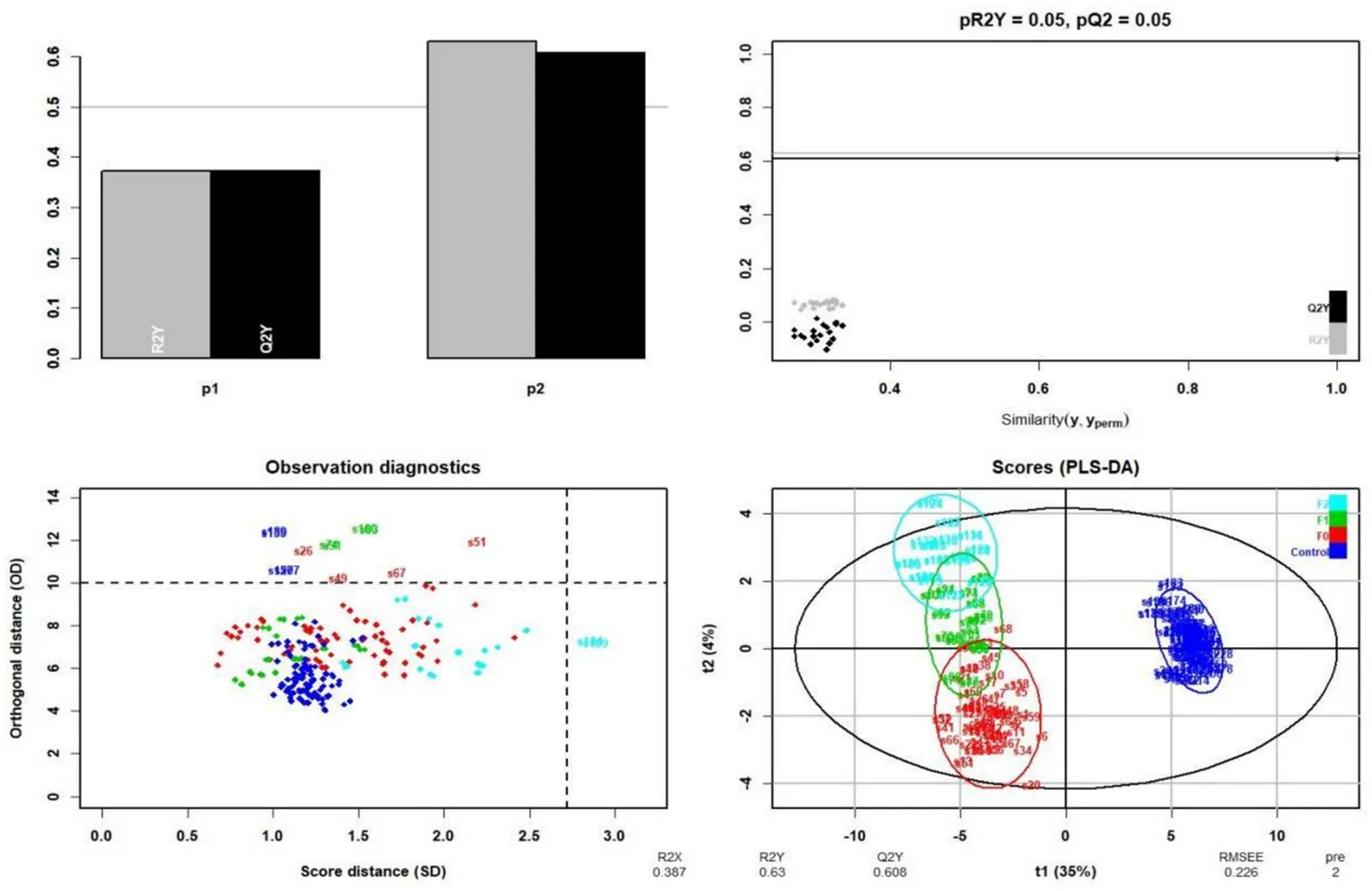
PLS-DA scores summarising the relationship between samples and their group classification (control, F0, F1, and F2), with model quality indicators (*R2X*, *R2Y*, and *Q2*) and permutation-based significance shown on the plot, alongside the observation diagnostics plot used to screen for outliers.

**Figure 5:**
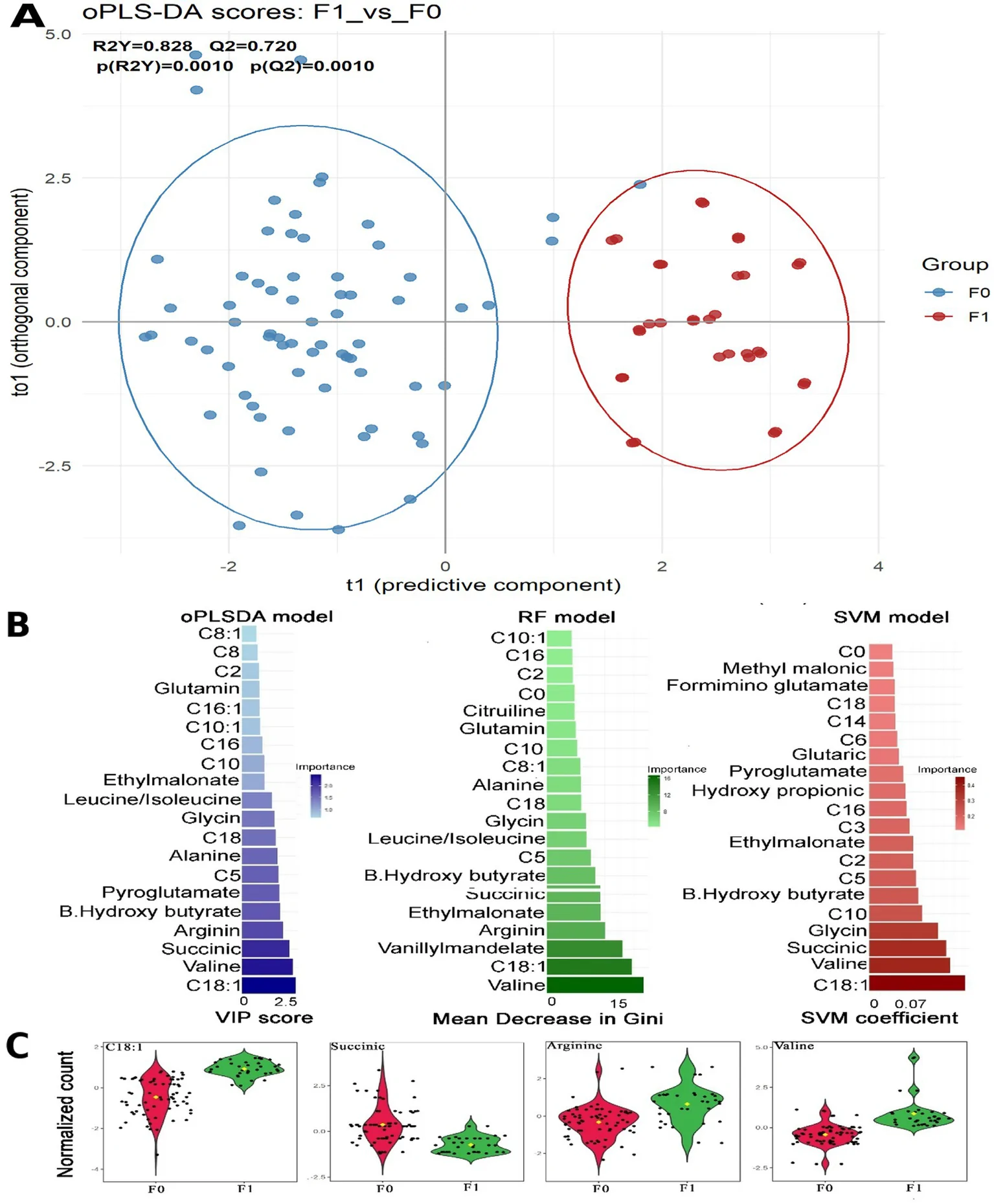
Metabolic differences between F0 and F1, using OPLS-DA, RF, and SVM. Panels **(A–B)** as in Figure 1; **(C)** Violin plots of representative discriminating metabolites (C18:1, succinic acid, arginine, and valine). This adjacent-stage contrast is the most demanding comparison and yields the most conservative validation metrics (Table 3).

To detect metabolites associated with MASLD severity, we first compared F0 and F1. PLS-DA separated the groups (Figure 4), and OPLS-DA held comparable results (R^2^X 0.151, R^2^Y 0.842, and Q^2^ 0.7, a realistic, non-saturated value for adjacent, metabolically similar stages). RF and SVM achieved clear separation (accuracies of 0.992 and 1.00, respectively; Table 3); RF across resampled test sets yielded similar values (Figure 5). Combining the three models with univariate analyses produced a list of 30 metabolites important for the F0–F1 contrast (9 organic acids, 7 amino acids, and 14 carnitines/acylcarnitines; Supplementary Figure S5).

#### 3.2.2 F0 versus F2

**Figure 6:**
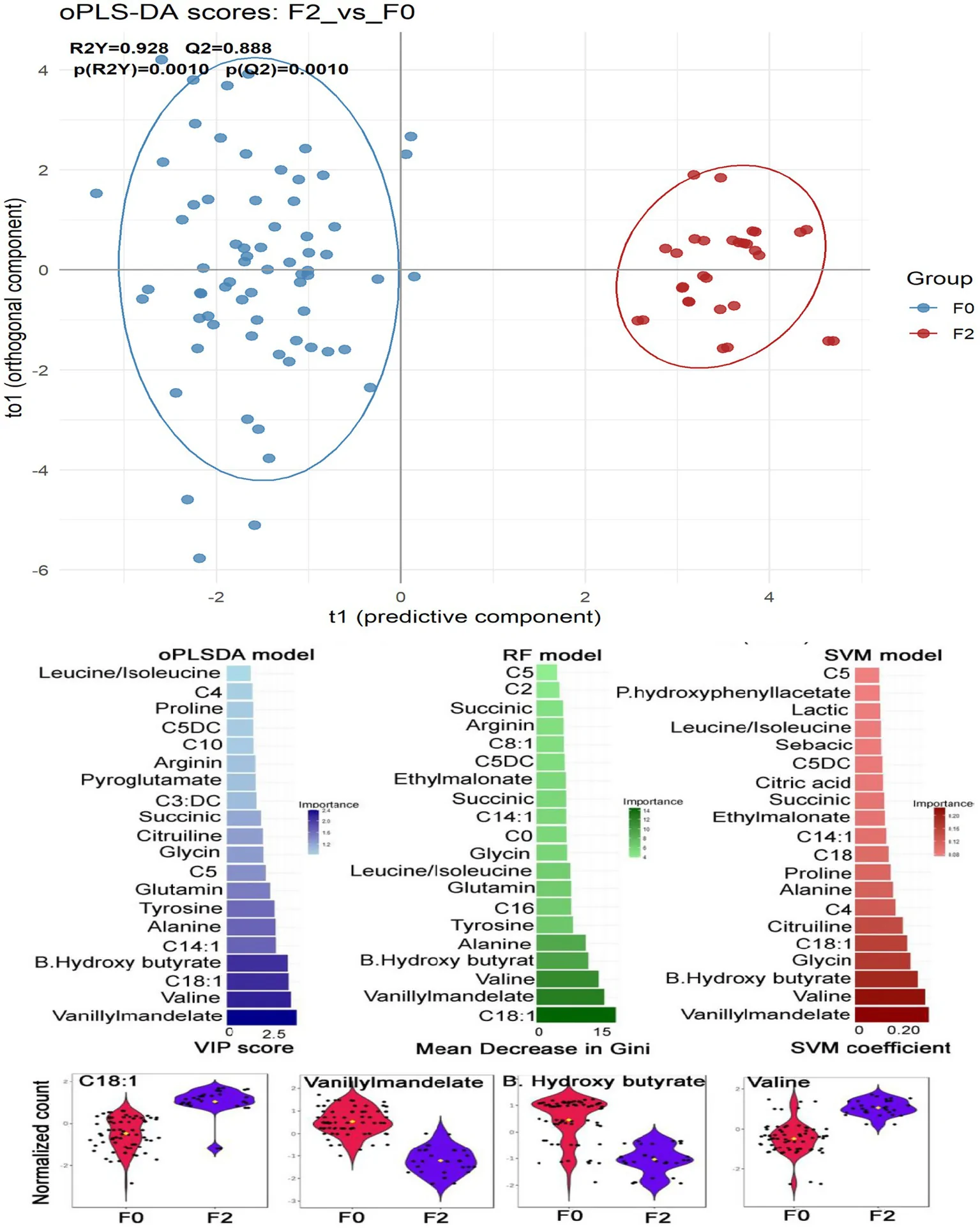
Metabolic differences between F0 and F2, using OPLS-DA, RF, and SVM. Panels **(A–B)** as in Figure 1; **(C)** violin plots of representative discriminating metabolites (C18:1, vanillylmandelate, β-hydroxybutyrate, and valine).

To identify metabolites associated with greater severity, F0 and F2 were compared. PLS-DA showed fair separation (Figure 4), while OPLS-DA gave high discrimination (R^2^X 0.207, R^2^Y 0.917, and Q^2^ 0.89). RF and SVM yielded significantly improved results (accuracies of 0.997 and 1.00; Figure 6). OPLS-DA returned 20 metabolites with VIP > 1, led by vanillylmandelate, valine, C18:1, and *β*-hydroxybutyrate. RF ranked C18:1, vanillylmandelate, valine, and β-hydroxybutyrate highest (Supplementary Figure S6). A 20-metabolite stage-stratification panel for F2 (Panel 2) was assembled from convergent OPLS-DA, SVM, and RF features, as well as univariate analyses. Pathway enrichment on these 20 metabolites identified alanine/aspartate/glutamate metabolism, glutathione metabolism, biosynthesis of unsaturated fatty acids, and valine/leucine/isoleucine biosynthesis as significantly enriched (Supplementary Figure S8).

### 3.3 Classification performance and model validation

To ensure robust, unbiased evaluation, we implemented a strict validation framework. The dataset was divided into training (70%) and independent test (30%) subsets by stratified sampling to preserve class balance. All preprocessing steps (KNN imputation and Z-score standardisation) and feature selection were performed exclusively on the training set, and the learned parameters were then applied to the test set, preventing data leakage.

#### 3.3.1 Diagnostic (case-discrimination) models: controls vs. F0, F1, and F2

For Controls vs. F0, the independent test set comprised 50 samples (30 controls and 20 F0); all three models (OPLS-DA, SVM, and RF) classified every test sample correctly, with accuracy, sensitivity, and specificity of 1.00 (95% CI: 0.93–1.00). For Controls vs. F1 (test set 30 controls + 10 F1) and Controls vs. F2 (test set 30 controls + 9 F2), all models achieved perfect classification (accuracy: 1.00; 95% CI: 0.91–1.00). These test-set numbers reconcile exactly to the 232-participant cohort (a 30% stratified split of 100 controls yields ~30 controls per comparison).

#### 3.3.2 Staging models: F0 vs. F1, F0 vs. F2

For F0 vs. F1 (test set 20 F0 + 10 F1) and F0 vs. F2 (test set 20 F0 + 9 F2), the models achieved excellent discrimination (accuracy 1.00; ROC AUC 1.00; Table 2). Because these test sets are small (*n* = 29–30), a point estimate of 1.00 carries a wide exact confidence interval (as wide as 0.90–1.00) and should be read as “excellent discrimination with wide uncertainty” rather than literal perfection; the cross-validated OPLS-DA Q^2^ for these metabolically matched comparisons is 0.72 and 0.89, respectively.

#### 3.3.3 Robustness testing

Repeated 10-fold cross-validation (10 repeats; 100 resampling iterations) for SVM and RF yielded mean ROC and sensitivity/specificity approaching 1.00, indicating stable performance irrespective of the training partition. Hyperparameter optimisation (cost/gamma for SVM; mtry for RF) was performed on the training data during cross-validation (as shown in the consolidated per-comparison validation figures).

Permutation testing (100 random label permutations for random forest, with the full pipeline including feature selection re-executed on each shuffle) produced empirical null distributions centred near chance, and the observed cross-validated performance fell entirely outside the null (empirical p ≈ 0.01, the resolution limit of a 100-permutation test); OPLS-DA permutation testing (1,000 permutations) gave permutation *p* ≤ 0.001 for R^2^Y and Q^2^ in every comparison. Because a leaking pipeline would also perform well on shuffled labels, this chance-level null is positive evidence that the classification reflects genuine biological structure rather than leakage or overfitting (consolidated per-comparison validation figures; Supplementary Table S4). Per-comparison permutation and ROC validation summaries for all three model families are provided in Supplementary Figures S9–S13.

#### 3.3.4 Comparison with established clinical indices

The metabolite-based models substantially exceeded standard non-invasive fibrosis scores in this cohort. For discriminating advanced fibrosis (*F* ≥ 2) from F0–F1, FIB-4 achieved an AUC of 0.74 (95% CI: 0.68–0.80) and APRI 0.70 (95% CI: 0.63–0.77), in line with a published systematic review (43). By contrast, the combined metabolite-ML models achieved excellent discrimination across all pairwise comparisons, and calibration was adequate for all models (Hosmer–Lemeshow *p* > 0.05; Supplementary Table S4), underscoring the added value of metabolomic profiling for early-stage differentiation (Table 2).

#### 3.3.5 Feature importance and consistency across algorithms

Key metabolites identified by VIP scores (OPLS-DA), mean decrease in Gini (RF), and SVM coefficients showed strong agreement, with citric acid, tiglylglycine, lactic acid, and several acylcarnitines (C14:1 and C18:1) consistently ranking highest (Figures 1, 5, 6). This convergence supports their biological relevance.

#### 3.3.6 Sensitivity analysis for metabolic confounding

After adjustment for BMI, HOMA-IR, and triglycerides, several metabolites remained significantly associated with disease status and fibrosis stage. For Controls vs. F0, lactic acid (OR: 4.2, 95% CI: 2.1–8.7, *p* < 0.001), citric acid (OR: 3.8, 95% CI: 1.9–7.5, *p* < 0.001), tiglylglycine (OR: 5.1, 95% CI: 2.4–10.9, *p* < 0.001), β-hydroxybutyrate (OR: 2.9, 95% CI: 1.4–6.1, *p* = 0.003), and C14:1 (OR: 3.2, 95% CI: 1.6–6.4, *p* = 0.001) remained significant. For F0 vs. F2, valine (OR: 4.5, 95% CI: 2.0–10.2, *p* < 0.001), C18:1 (OR: 3.9, 95% CI: 1.8–8.5, *p* < 0.001), and vanillylmandelate (OR: 0.3, 95% CI: 0.1–0.7, *p* = 0.005) were robust after adjustment. Full results appear in Supplementary Table S3.

## 4 Discussion

Metabolomic analysis is well positioned to help unravel the pathophysiology of MASLD, a multifaceted condition shaped by interactions between MetS, liver disease, and other organs and influenced by heritable and environmental factors (7). MASLD affects approximately 30% of adults worldwide and is an increasingly important cause of liver-related morbidity and mortality (2, 3). In this study, we sought to determine whether metabolic reprogramming in MASLD is associated with disease severity (fibrosis stages F0–F2), to identify candidate diagnostic markers (for distinguishing MASLD from controls) and stage-stratification markers, and to inform stage-specific interpretation (23).

Because this study is cross-sectional, the terms “diagnostic” and “stage-stratification” refer to the ability to discriminate between groups (healthy vs. MASLD) and between prevalent fibrosis stages, respectively, rather than to predict future clinical outcomes. Prospective validation is required to establish true prognostic value.

We integrated three machine learning models, namely, OPLS-DA, RF, and SVM, with univariate analysis to develop a robust analytical framework. Although OPLS-DA performed well, the non-linear RF and SVM models were associated with superior ability to capture complex data structure (Table 3). RF is well-suited to biological data because its non-linear algorithm can represent intricate relationships (47), and both RF and SVM are relatively robust to outliers and overfitting (37). This aligns with advances in machine learning applications for liver disease (48): The European NAFLD Registry study reported that XGBoost could reach 89.9% accuracy in identifying patients at risk of MASH using routine clinical variables (48), and Zhu et al. demonstrated that simple demographic and clinical variables could achieve robust prediction through appropriate algorithms (49, 50).

There remains no reliable method for early detection of MASLD; liver enzymes may be normal (17), and prediction scores have yielded mixed findings (18–21). Identifying patients at risk is crucial for the rational allocation of limited resources (35), particularly as several liver-specific therapies approach clinical use, and reliable tools are needed to monitor disease and act as companion diagnostics (7). To our knowledge, this dual-matrix targeted metabolomic study is among the first to combine blood and urinary profiling with three machine learning algorithms for stage-specific MASLD biomarker discovery.

### 4.1 Metabolites associated with early-stage MASLD

Controls were clearly distinguishable from MASLD patients by metabolite profile, consistent with a distinct metabolic signature associated with disease presence. Pairwise comparisons between consecutive stages suggested that metabolome alterations may occur early, before severe disease develops. The case-discrimination panel (Panel 1) comprised C18:1, β-hydroxybutyrate, adipic acid, C0, C10:1, C14:1, C2, C3, citric acid, formiminoglutamate, glucose, glycine, lactic acid, Leu/Ile, pyroglutamate, sebacic and suberic acids, tiglylglycine, and valine candidate markers associated with disease presence.

MASLD is characterised by altered hepatocyte metabolism, including increased gluconeogenesis and lactate production, and altered TCA cycle activity, alongside reduced ketone body production and mitochondrial ATP synthesis (51). β-Hydroxybutyrate, the most abundant ketone body (45), reflects fatty acid metabolism abnormalities and may be associated with early hepatic fibrosis (31). Reduced urinary formiminoglutamate and vanillylmandelate, both linked to MetS, may signal early hepatic pathology (46).

Acylcarnitines are key FAO intermediates: Fatty acids are converted to acyl-CoA and then to acylcarnitine via CPT1A, with CPT2 regenerating free carnitine and acyl-CoA after mitochondrial import. MASLD patients exhibit elevated serum acylcarnitine levels (26). Oleoylcarnitine (C18:1) has been described as an onco-metabolite promoting stemness in hepatocellular carcinoma via STAT3 (52), and altered acylcarnitine composition may be associated with MASLD progression. Our findings are consistent with prior reports of accumulated long-chain and diminished short-chain acylcarnitines in NASH (53, 54) and elevated C18:1 in NASH (54), and with long-chain acylcarnitines relating to NAFLD occurrence (55). Medium- and long-chain acylcarnitines can trigger pro-inflammatory signalling in monocytes and stimulate secretion of TNF-*α*, IL-6, and MCP-1 (56, 57), processes implicated in MASLD (26). In contrast, L-carnitine supplementation reduces inflammation and oxidative stress (51, 58).

The enriched pathways associated with these markers included glutathione and glyoxylate/dicarboxylate metabolism, as well as valine/leucine/isoleucine biosynthesis. Glutathione supports phase-II detoxification (59), and phase-II dysfunction may exacerbate oxidative stress and liver injury (60); the glyoxylate shunt supplies carbon skeletons for biosynthesis and gluconeogenesis (61). These associations suggest that abnormalities in phase-II detoxification and fatty acid metabolism may indicate early fibrotic change in MASLD. However, further work is needed to confirm their diagnostic utility across the disease spectrum (31).

### 4.2 Amino acid metabolism

BCAAs (leucine, isoleucine, and valine) are essential amino acids with anabolic effects and are central to protein synthesis, glucose metabolism, and mTOR and PI3K signalling (62, 63). BCAA catabolism is reduced in early or moderate MASLD, raising serum BCAAs and reflecting diminished mitochondrial metabolism (64). Kakazu et al. reported that plasma BCAAs correlated with hepatocyte lipid-droplet heterogeneity in NAFLD/NASH via SREBP-1 and p70S6 kinase (65). Our findings of elevated valine and Leu/Ile, together with glutamate and alanine and reduced glycine in moderate fibrosis, are consistent with previous studies of metabolic disease, including MASLD (66–68) and its association with T2DM (69); IR and mitochondrial dysfunction are proposed as shared pathways (67, 70). As liver disease worsens, exaggerated muscle amino acid catabolism from hyperammonaemia can instead lower BCAAs, and BCAA supplementation in cirrhosis reduces serum glucose. These observations suggest that dietary amino acid and BCAA measures may be candidate biomarkers for MASLD, irrespective of MetS severity (71, 72).

Glutamine, the most abundant amino acid, is synthesised from glutamate and ammonia by glutamine synthetase; it detoxifies ammonia in the liver and maintains acid–base balance in the kidney (73, 74). Liver histology has most closely linked glutamate to the degree of fibrosis (75). Glutathione metabolism is altered in MASLD, probably reflecting increased turnover under oxidative stress from mitochondrial dysfunction (76), with reduced glutathione, glycine, and serine and increased glutamate, pyroglutamate, cysteine, and homocysteine (75, 77); increases in glutamate, pyroglutamate, and the glutamate-to-glutamine ratio are associated with MASH (68, 72). Blood glutamate and glycine may thus be associated with liver fibrosis, independent of IR (71, 77), and glutamine may be associated with fibrotic recovery (78).

### 4.3 Fatty acid metabolism

Hepatic lipidome alterations in MASLD generally parallel plasma changes. Puri et al. observed a progressive decline in n-6 and n-3 PUFAs and a rising n-6:n-3 ratio from controls to steatosis to MASH (79). Monounsaturated and free saturated fatty acids are lipotoxic and disproportionately elevated in MASLD (80). Long-chain ceramides (C16:0, C18:0) generated by CerS6 are closely associated with IR, and obesity, a MASLD risk factor (41), is linked to increased CerS6 expression; ceramides can impair insulin signalling in a positive-feedback loop (81). Butyrate is often converted to ethylmalonate (82), and elevated butyryl-CoA from isoleucine catabolism is linked to ethylmalonic aciduria (31, 83); as fatty acid breakdown yields *β*-hydroxybutyrate (84), fatty acid metabolism abnormalities may be associated with hepatic fibrosis (31). Succinate, a TCA intermediate with antilipolytic action, is exported from mitochondria via the dicarboxylate carrier and modulates hepatic lipid handling and adipocyte lipolysis (85); an inverse association between succinate and hepatic steatosis has been reported (31). Malonate contributes to fatty acid synthesis via malonyl-CoA and competitively inhibits succinate dehydrogenase (86, 87); its role in the gut–liver axis in MASLD remains uncertain, although high-fat-diet models show increased faecal malonate (88).

### 4.4 Urine-based metabolomics

Urine is a valuable, non-invasive source of biomarkers (27, 89), less affected by sampling time and diurnal variation, and informative about ongoing metabolism (28). Urinary organic acid testing is established for metabolic disease and inborn errors of metabolism (29, 30) and reveals intermediates relevant to steatosis or fibrosis (31). Urinary metabolites may carry predictive value (7): MASLD subtypes differ in PI3K–Akt signalling, carbohydrate and cholesterol metabolism, and inflammatory responses (90); 31 urinary metabolites have been reported to distinguish MASH from MASLD, largely affecting amino acids and pentose-phosphate-linked nucleic acids (37); and sebacic and arachidonic acids and pregnenolone sulphate have been used to differentiate lean- from obese-NAFLD (91). Urinary profiling may also track therapy: Metabolic changes in tyrosine/valine metabolism, pseudouridine, and 2-hydroxyisobutyrate were noted in children receiving probiotic medicinal food (92), supporting the use of urine biomarkers as non-invasive surrogates for assessing liver disease.

### 4.5 Metabolites associated with MASLD severity, and a testable hypothesis

*A synthesis and hypothesis*. Beyond cataloguing individual metabolites, our data reveal a coherent signature as fibrosis advances within the MASLD population: (i) progressive accumulation of long-chain acylcarnitines (C18:1 and C14:1 rising, short-chain C5 falling), indicating a bottleneck at the CPT2/*β*-oxidation step; (ii) rising branched-chain amino acids (valine and Leu/Ile), consistent with suppressed BCKDH-mediated catabolism; and (iii) falling urinary vanillylmandelate, indicating reduced sympatho-adrenal catecholamine turnover. We propose a specific, testable model that early fibrogenesis in MASLD is driven by a primary insufficiency of mitochondrial β-oxidation that precedes and then promotes lipotoxic stellate-cell activation, rather than merely arising as a downstream consequence of established mitochondrial injury. In this model, long-chain acylcarnitine “overflow” is an early, quantifiable readout of the β-oxidation bottleneck; the BCAA rise reflects competition for shared, overloaded mitochondrial oxidative capacity; and C18:1 itself, shown to act as a pro-inflammatory and pro-tumorigenic lipid via STAT3, provides a plausible molecular link from the metabolic lesion to inflammation and fibrogenesis. Because our cross-sectional data cannot resolve direction (cause versus consequence), this hypothesis should be tested by longitudinal sampling to establish temporal ordering, stable-isotope flux studies of hepatic β-oxidation, and controlled modulation of CPT2 activity.

The stage-stratification panel (Panel 2) comprised vanillylmandelate, citrulline, C18:1, succinate, 3-hydroxybutyrate, pyroglutamate, C5, alanine, Leu/Ile, C10, C16, glutamine, C2, C8:1, C0, glycine, C18, ethylmalonate, and valine and was associated with MASLD severity. Enriched pathways included alanine/aspartate/glutamate metabolism, glutathione metabolism, biosynthesis of unsaturated fatty acids, and valine/leucine/isoleucine biosynthesis. MASLD is associated with increased total free fatty acids (93) and a higher PUFA-to-total-FFA ratio has been reported to be inversely associated with MASLD (94).

These enriched pathways, namely, glutathione metabolism, BCAA biosynthesis, TCA-cycle intermediates, and unsaturated fatty acid synthesis, are the pathways modulated by emerging MASLD therapeutics. Semaglutide reduces hepatic steatosis partly via suppression of *de novo* lipogenesis and improved BCAA catabolism, mechanisms reflected in our case-discrimination panel (95), and resmetirom (a thyroid hormone receptor-β agonist) targets hepatic fatty acid oxidation, as reflected in our acylcarnitine signature (C14:1 and C18:1) (96). The identified panels may therefore also serve as candidate pharmacodynamic biomarkers to monitor treatment response in future trials.

Acylcarnitines, as β-oxidation intermediates, increased progressively in serum as fibrosis advanced, whereas free carnitine did not (26). A prediction signature of decreased C5:0 with elevated C14:1 and C18:1 has been associated with MASLD progression (52), with reports of increased short- and long-chain acylcarnitines (54) and higher acylcarnitines in MASH than in MASLD (97). Together, these observations point to increasingly impaired β-oxidation in MASLD (7).

### 4.6 Clinical translation and comparative performance

Urinary and blood metabolite analysis can be performed on established analytical platforms available in most clinical laboratories. The European NAFLD Registry study showed machine learning models using routine clinical variables could achieve high accuracy (48), while our approach adds mechanistic insight through metabolite profiling. Combined with the demographic-based screening of Zhu et al. (50), sophisticated metabolomic profiling could refine risk assessment in individuals requiring detailed evaluation. Our metabolomic models outperformed traditional clinical markers (FIB-4, APRI) in this cohort, reflecting the closer relationship between metabolites and functional disease processes (98).

*A parsimonious, deployable subset*. The 20-metabolite panels are discovery-phase signatures that map the breadth of metabolic reprogramming; they are not intended for wholesale clinical use. Across all three algorithms, and after adjustment for BMI, HOMA-IR, and triglycerides (Supplementary Table S3), a compact core carries most of the signal: for case detection, lactic acid, citric acid, tiglylglycine, β-hydroxybutyrate, and C14:1; for stage stratification, valine, C18:1, and vanillylmandelate. Such a three- to five-analyte panel is realistic for routine use. Notably, MS/MS of dried-blood-spot acylcarnitines and amino acids and GC/MS of urinary organic acids are everyday workhorses in newborn-screening and inborn-error laboratories: high-throughput, inexpensive, and dried-blood-spot compatible, so a tiered strategy (an inexpensive first-line subset, with the fuller panel reserved for confirmation) is feasible.

### 4.7 Methodological considerations and framing

Strengths include the use of three complementary machine learning algorithms, comprehensive pathway analysis, and a strictly leakage-free validation framework with permutation testing and repeated cross-validation. The consistent identification of key metabolites across models strengthens confidence in their relevance. The focus on early-stage MASLD (F0–F2) addresses a genuine clinical need, in contrast to many studies of advanced disease.

We emphasise, however, that these findings are exploratory and hypothesis-generating. This cross-sectional discovery establishes associations with disease presence and prevalent fibrosis stage; it does not establish prognosis or clinical utility. Translation will require prospective prognostic validation, external validation in metabolically matched cohorts, a parsimonious deployable assay, and formal decision-curve analysis, as detailed below.

## 5 Limitations and future directions

Several limitations must be acknowledged. First and most importantly, the case-detection comparison contrasts lean controls with obese MASLD patients, so the covariate distributions overlap poorly and the positivity assumption is weak; statistical adjustment alone cannot fully isolate a liver-specific effect. Accordingly, the case-detection findings are presented as exploratory case–control discrimination rather than a validated diagnostic. Crucially, however, the fibrosis-stage comparisons (F0 vs. F1, F0 vs. F2) are performed entirely within the MASLD population, where the groups are metabolically matched (median BMI of 30–32 kg/m^2^ with overlapping IQRs), so the lean-versus-obese contrast does not account for the staging biomarkers.

Cardiometabolic risk factors inherent to MASLD (T2DM, obesity, and dyslipidaemia) can independently alter the metabolites studied; for example, BCAAs are elevated in T2DM and IR (99), and medium- and long-chain acylcarnitines are elevated in obesity independent of fibrosis stage (57). Although our models included insulin and HOMA-IR, and we performed a sensitivity analysis adjusting for BMI, HOMA-IR, and triglycerides, these adjustments were only partial, and formal mediation analyses separating liver-specific from systemic contributions were not performed. The absence of a comparator group with obesity/metabolic syndrome but without hepatic steatosis prevents definitive attribution of all differences to MASLD-specific pathology; future studies should include such a matched arm and stratify by T2DM status, BMI category, and medication use.

Case ascertainment used ultrasound, which is insensitive for minimal steatosis (< 5–20%); any resulting misclassification would bias associations towards the null, making our significant findings conservative. Controls were screened with ultrasonography, liver function tests, and viral/autoimmune serology, but occult minimal steatosis cannot be fully excluded.

Because the study is cross-sectional, the stage-stratification panel’s true prognostic value requires validation in prospective, longitudinal cohorts. The design was powered to discriminate fibrosis stages (F0–F2) rather than steatosis grades. However, CAP data were recorded, and a formal CAP-stratified analysis was underpowered and is a priority for the next phase. Finally, external validation in independent, multi-ethnic, metabolically diverse cohorts with comparators matched for BMI, metabolic-syndrome status, diabetes, age, and sex is required before clinical application, and formal decision-curve analysis and quantification of incremental value over clinical variables remain to be performed.

## 6 Conclusion

This exploratory analysis underscores the utility of multivariate metabolomic approaches in MASLD. We identified a case-discrimination panel (*β*-hydroxybutyrate, adipic acid, acylcarnitines [C0, C2, C3, C10:1, C14:1, and C18:1], formiminoglutamate, glucose, glycine, citric and lactic acids, Leu/Ile, pyroglutamate, sebacic and suberic acids, tiglylglycine, and valine) and a stage-stratification panel (valine, ethylmalonate, glycine, acylcarnitines [C0, C8:1, C2, C5, C16, C10, C18, and C18:1], Leu/Ile, alanine, glutamine, pyroglutamate, 3-hydroxybutyrate, succinate, vanillylmandelate, and citrulline) associated with fibrosis stage in established MASLD. Machine learning can contribute to the discovery of candidate biomarkers associated with liver-related pathology; however, these findings are exploratory, and prospective, externally validated studies with metabolically matched comparators are required before clinical application.

## Data Availability

The processed, de-identified metabolite dataset supporting the conclusions of this article will be deposited in a link in a Supplementary Tables S1, S3; the data and code are available from the corresponding author, SA (shimaa.abdelsattar@liver.menofia.edu.eg) or author MN (Mahmoud.Nazih5698@pharm.aun.edu.eg), upon reasonable request.
