# Supplementary material for: Machine learning-based identification of targeted metabolomic biomarkers for early diagnosis and fibrosis-stage discrimination in metabolic dysfunction-associated steatotic liver disease
Source: Front Nutr. 2026 Aug 24;13:1849329. doi: 10.3389/fnut.2026.1849329 (PMC13547064; doi:10.3389/fnut.2026.1849329)
Supplement: Supplementary file 1 [file Table_1.DOCX]

. All preprocessing procedures, including missing value imputation using K-nearest neighbors (KNN) and data standardization (Z-score scaling), were performed exclusively on the training dataset. The learned preprocessing parameters were subsequently applied to the independent test set. This procedure ensures that no information from the test set was used during model training and therefore eliminates potential data leakage.

Model performance was evaluated using independent test datasets that were not used during model training. For the Control vs F0 comparison, the independent test set consisted of 50 samples (30 controls and 20 F0 cases). For the Control vs F1 comparison, the independent test set included 40 samples (30 controls and 10 F1 cases). For the Control vs F2 comparison, the independent test set consisted of 39 samples (30 controls and 9 F2 cases). In all three analyses, the models correctly classified all independent test samples, resulting in an accuracy of 1.0 with sensitivity and specificity of 1.0. The corresponding 95% confidence intervals for the accuracy were 0.93–1.00 for the Control vs F0 comparison and 0.91–1.00 for both the Control vs F1 and Control vs F2 comparisons, indicating statistical stability of the estimates.

To further evaluate model robustness during training, repeated 10-fold cross-validation (10 repeats) was performed for the Support Vector Machine model. Across 100 resampling iterations, the model maintained consistently high performance, with mean ROC values close to 1.0 and sensitivity and specificity approaching 1.0, demonstrating stable predictive performance across multiple training partitions. In addition, permutation testing was conducted to determine whether the observed classification accuracy could arise from random correlations. Class labels were randomly shuffled 500 times and the classification models were retrained for each permutation. The resulting permutation accuracy distribution ranged approximately from 0.30 to 0.80 with a mean around 0.55, consistent with random classification. In contrast, the observed model accuracy was 1.0, which lies far outside the permutation distribution (empirical p < 0.002; 0 of 500 permutations ≥ observed accuracy), indicating that the model captures genuine biological structure rather than random patterns.

Importantly, three independent classification approaches—OPLS-DA, Support Vector Machine, and Random Forest—produced consistent results across all comparisons (Control vs F0, Control vs F1, and Control vs F2). All three algorithms achieved identical classification of the independent test samples. The agreement among multiple machine learning algorithms further supports the robustness of the metabolomic signatures distinguishing the groups. Furthermore, the probability distributions of predicted class probabilities showed clear separation between groups, with Control samples assigned probabilities close to 1 and fibrosis samples assigned probabilities close to 0, demonstrating strong discriminative capacity of the metabolite panel. Receiver operating characteristic (ROC) analysis further confirmed the discriminative performance of the models, with both Support Vector Machine and Random Forest achieving an area under the curve (AUC) of 1.0 on the independent test datasets. Taken together, the independent test prediction, repeated cross-validation, permutation testing, and agreement among multiple machine learning algorithms demonstrate that the observed classification performance is robust and unlikely to result from overfitting or information leakage. These analyses confirm that the metabolomic signatures distinguishing Control samples from fibrosis stages F0, F1, and F2 are statistically robust and reproducible.

Figure S1	Model performance and validation metrics of control and F0 sample groups

| **Model** | **Training Samples** | **Variables** | **R²X** | **R²Y** | **Q²** | **Accuracy** | **Sensitivity** | **Notes** |
| --- | --- | --- | --- | --- | --- | --- | --- | --- |
| OPLS-DA | 118 | 51 | 0.376 | 0.978 | 0.971 | 1.00 | 1.00 | permutation pR^2^Y = 0.01 |
| SVM | 118 | 51 | – | – | – | 1.00 | 1.00 | 10-fold CV |
| RF | 118 | 51 | – | – | – | 1.00 | 1.00 | 500 trees |
| **Model** | **Specificity** | **Balanced accuracy** | **95% CI** | **ROC** | Kappa | **RMSEE** | *P-*Value [Acc > NIR] |  |
| OPLS-DA | 1.00 | 1.00 | 0.9289-1 | 1.00 | 1.00 | 0.074 | 8.083e-12 | permutation pQ2 = 0.01 |
| SVM | 0.977-0.98 | 1.00 | 0.9289-1 | 1.00 | 1.00 | - | 8.083e-12 |  |
| RF | 1.00 | 1.00 | 0.9289-1 | 1.00 | 1.00 | - | 8.083e-12 |  |
|  |  |  |  |  |  |  |  |  |

Figure S2 Model performance and validation metrics of control and F1 sample groups

| **Model** | **Training Samples** | **Variables** | **R²X** | **R²Y** | **Q²** | **Accuracy** | **Sensitivity** | **Notes** |
| --- | --- | --- | --- | --- | --- | --- | --- | --- |
| OPLS-DA | 94 | 51 | 0.406 | 0.985 | 0.975 | 1.00 | 1.00 | permutation pR^2^Y = 0.01 |
| SVM | 94 | 51 | – | – | – | 1.00 | 1.00 | 10-fold CV |
| RF | 94 | 51 | – | – | – | 1.00 | 1.00 | 500 trees |
| **Model** | **Specificity** | **Balanced accuracy** | **95% CI** | **ROC** | Kappa | **RMSEE** | *P-*Value [Acc > NIR] |  |
| OPLS-DA | 1.00 | 1.00 | 0.9119-1 | 1.00 | 1.00 | 0.0551 | 1.006e-05 | permutation pQ2 = 0.01 |
| SVM | 0.958-0.955 | 1.00 | 0.9119-1 | 1.00 | 1.00 | - | 1.006e-05 |  |
| RF | 1.00 | 1.00 | 0.9119-1 | 1.00 | 1.00 | - | 1.006e-05 |  |
|  |  |  |  |  |  |  |  |  |

Figure S3 Model performance and validation metrics of control and F2 sample groups

| **Model** | **Training Samples** | **Variables** | **R²X** | **R²Y** | **Q²** | **Accuracy** | **Sensitivity** | **Notes** |
| --- | --- | --- | --- | --- | --- | --- | --- | --- |
| OPLS-DA | 91 | 51 | 0.417 | 0.977 | 0.966 | 1.00 | 1.00 | permutation pR^2^Y = 0.01 |
| SVM | 91 | 51 | – | – | – | 1.00 | 1.00 | 10-fold CV |
| RF | 91 | 51 | – | – | – | 1.00 | 1.00 | 500 trees |
| **Model** | **Specificity** | **Balanced accuracy** | **95% CI** | **ROC** | Kappa | **RMSEE** | *P-*Value [Acc > NIR] |  |
| OPLS-DA | 1.00 | 1.00 | 0.9097-1 | 1.00 | 1.00 | 0.064 | 3.599e-05 | permutation pQ2 = 0.01 |
| SVM | 1.00 | 1.00 | 0.9097-1 | 1.00 | 1.00 | - | 3.599e-05 |  |
| RF | 1.00 | 1.00 | 0.9097-1 | 1.00 | 1.00 | - | 3.599e-05 |  |
