# Supplementary material for: Machine learning-based identification of targeted metabolomic biomarkers for early diagnosis and fibrosis-stage discrimination in metabolic dysfunction-associated steatotic liver disease
Source: Front Nutr. 2026 Aug 24;13:1849329. doi: 10.3389/fnut.2026.1849329 (PMC13547064; doi:10.3389/fnut.2026.1849329)
Supplement: Supplementary file 2 [file Table_2.DOCX]

 Table S1”:.  *complete ranges of variables,*  *“Ranges are presented as minimum – maximum values observed in each group.” in table 1*

| **Variable** | **F0 (n=68)** | **F1 (n=34)** | **F2 (n=30)** | **Controls (n=100)** |
| --- | --- | --- | --- | --- |
| **Glucose (mg/dL)** | 76 – 99 | 79 – 97 | 80 – 97 | 73 – 91 |
| **BMI (kg/m²)** | 27.1 – 33.0 | 29.6 – 33.8 | 30.2 – 34.0 | 20.6 – 22.4 |
| **Waist circumference (cm)** | 97.9 – 110.9 | 95.2 – 110.1 | 98.9 – 111.5 | 66.2 – 71.9 |
| **Hip circumference (cm)** | 108.5 – 116.2 | 100.7 – 122.0 | 108.0 – 129.4 | 84.8 – 94.4 |
| **Waist‑to‑hip ratio** | 0.88 – 0.98 | 0.86 – 1.00 | 0.83 – 0.98 | 0.74 – 0.80 |
| **Systolic BP (mmHg)** | 110 – 135 | 110 – 140 | 110 – 140 | 100 – 125 |
| **Diastolic BP (mmHg)** | 70 – 90 | 70 – 100 | 80 – 90 | 70 – 80 |

# Table S2 a . Plasma Amino Acid and organic acids Profiles Among MASLD Stages and Controls

| **Variable** | **F0**  **N=68** | **F1**  **N=34** | **F2**  **N=30** | **Controls**  **N=100** | **p-value** | **Post-hoc test** |
| --- | --- | --- | --- | --- | --- | --- |
| Glutaric | 3.73 (1.85) | 3.59 (2.22) | 3.58 (2.31) | 3.645 (1.88) | 0.3 | NA |
| Adipic | 3.79 (1.21) | 3.75 (1.71) | 3.595 (1.07) | 2.19 (0.94) | <0.001 | ^a, b, c^ |
| Pimelic | 1.63 (0.47) | 1.64 (0.47) | 1.655 (0.82) | 1.63 (0.11) | 0.83 | NA |
| Suberic | 1.59 (1.04) | 1.65 (1.09) | 1.875 (0.70) | 1.57 (0.18) | <0.001 | ^a, b, c^ |
| Sebacic | 4.42 (4.11) | 4.8 (4.12) | 1.63 (3.12) | 0.92 (0.00) | <0.001 | ^a, b, c^ |
| Methylmalonic | 4.69 (3.31) | 5.52 (2.86) | 4.575 (3.71) | 3.8 (1.59) | <0.001 | ^a, b, c^ |
| Ethylmalonate | 2.82 (1.84) | 1.59 (0.84) | 1.555 (1.08) | 3.76 (1.18) | <0.001 | ^a, b, c, d, e^ |
| Succinic | 4.4 (1.95) | 3.0 (1.00) | 3.4 (1.10) | 4.545 (1.48) | <0.001 | ^b, c, d, e^ |
| Citric Acid | 13.5 (2.42) | 13.8 (3.00) | 14.0 (3.30) | 3.57 (2.40) | <0.001 | ^a, b, c^ |
| Hydroxypropionic | 7.2 (2.00) | 6.4 (1.95) | 6.45 (1.70) | 3.565 (2.98) | <0.001 | ^a, b, c^ |
| Tiglylglycine | 3.92 (1.00) | 3.92 (1.90) | 3.92 (1.90) | 0.795 (0.29) | <0.001 | ^a, b, c^ |
| Lactic | 23.5 (6.45) | 23.5 (9.90) | 23.0 (2.40) | 3.165 (1.17) | <0.001 | ^a, b, c^ |
| ?-hydroxybutyrate | 7.1 (1.10) | 5.27 (0.69) | 4.375 (1.02) | 2.555 (0.55) | <0.001 | ^a, b, c, e^ |
| Vanillylmandelate | 2.1 (0.30) | 1.5 (0.35) | 1.4 (0.40) | 2.555 (0.63) | <0.001 | ^a, b, c, d, e^ |
| Pyroglutamate | 19.0 (3.00) | 17.0 (3.50) | 17.0 (3.00) | 24.205 (2.99) | <0.001 | ^a, b, c^ |
| p-hydroxyphenyllactate | 0.25 (0.11) | 0.24 (0.10) | 0.315 (0.18) | 0.21 (0.10) | <0.001 | ^a, b, c^ |
| Formiminoglutamate | 0.19 (0.12) | 0.17 (0.10) | 0.185 (0.09) | 0.65 (0.48) | <0.001 | ^a, b, c^ |
| ASP (ng/mL) | 180.33 (109.78) | 180.00 (29.50) | 182.93 (61.95) | 49.00 (18.70) | <0.00 | ^a, b, c^ |
| CIT:Phe | 0.38 (0.16) | 0.44 (0.17) | 0.25 (0.05) | 0.33 (0.53) | <0.00 | ^a, b, c, f^ |
| Citrulline | 27.50 (5.70) | 28.80 (4.82) | 21.34 (2.30) | 19.20 (9.82) | <0.00 | ^a, b, e, f^ |
| Glutamine | 130.50 (45.45) | 117.50 (23.06) | 195.25 (65.01) | 93.50 (41.02) | <0.00 | ^a, b, c, e, f^ |
| Glycine | 175.00 (48.24) | 155.50 (17.08) | 150.28 (14.44) | 207.00 (54.25) | <0.00 | ^a, b, c, d^ |
| Leu/Ile | 124.00 (23.10) | 134.50 (8.28) | 135.85 (7.55) | 82.45 (20.00) | <0.00 | ^a, b, c, e^ |
| Methionine | 24.17 (2.85) | 24.96 (1.64) | 24.98 (2.74) | 25.00 (2.00) | 0.05 | ^NA^ |
| Ornithine | 80.21 (16.00) | 80.45 (19.00) | 87.47 (33.00) | 100.00 (16.00) | <0.00 | ^a, b, c^ |
| Proline | 104.40 (36.20) | 110.18 (35.10) | 95.35 (15.03) | 90.40 (30.52) | <0.00 | ^a, b^ |
| Phenylalanine | 54.14 (6.47) | 57.00 (4.95) | 54.69 (3.42) | 54.00 (3.00) | <0.00 | ^b^ |
| Tyrosine | 50.00 (9.38) | 50.70 (6.17) | 58.79 (4.26) | 48.00 (5.38) | <0.00 | ^a, b, c, e, f^ |
| Valine | 75.50 (6.88) | 84.00 (5.18) | 86.15 (3.87) | 66.17 (16.96) | <0.00 | ^a, b, c, d, e^ |
| Alanine | 305.30 (32.94) | 320.19 (28.73) | 347.35 (27.80) | 290.00 (53.50) | <0.00 | ^b, c, d, e^ |
| Arginine | 8.36 (2.50) | 11.32 (2.71) | 9.44 (3.27) | 6.00 (3.92) | <0.00 | ^a, b, c, d^ |
| Superscript letters indicate significant post hoc comparisons (p < 0.05): a = F0 vs. Control; b = F1 vs. Control; c = F2 vs. Control; d = F0 vs. F1; e = F0 vs. F2; f=F1 vs. F2. | | | | | | |

| **Variable** | **F0**  **N=68** | **F1**  **N=34** | **F2**  **N=30** | **Controls**  **N=100** | **p-value** | **Post-hoc test** |
| --- | --- | --- | --- | --- | --- | --- |
| C0 | 21.50 (11.58) | 29.24 (9.81) | 29.32 (5.40) | 13.20 (4.65) | <0.00 | ^a, b, c^ |
| C2 | 8.45 (3.00) | 9.56 (3.00) | 9.06 (1.00) | 5.00 (2.00) | <0.00 | ^a, b, c^ |
| C3 | 0.76 (0.34) | 0.91 (0.25) | 0.86 (0.19) | 0.34 (0.25) | <0.00 | ^a, b, c^ |
| C4 | 0.15 (0.06) | 0.14 (0.07) | 0.17 (0.03) | 0.11 (0.05) | <0.00 | ^a, c, f^ |
| C5 | 0.14 (0.04) | 0.12 (0.03) | 0.12 (0.02) | 0.12 (0.14) | <0.00 | ^d, e^ |
| C5 DC | 0.06 (0.02) | 0.08 (0.03) | 0.09 (0.04) | 0.12 (0.03) | <0.00 | ^a, b, e^ |
| C6 | 0.03 (0.03) | 0.04 (0.02) | 0.04 (0.03) | 0.03 (0.01) | <0.00 | ^b, c^ |
| C8 | 0.03 (0.03) | 0.04 (0.02) | 0.04 (0.02) | 0.03 (0.01) | 0.27 | NA |
| C10 | 0.07 (0.06) | 0.07 (0.03) | 0.06 (0.03) | 0.04 (0.06) | <0.00 | ^a, b^ |
| C12 | 0.02 (0.04) | 0.02 (0.02) | 0.02 (0.02) | 0.02 (0.01) | 0.21 | ^NA^ |
| C14 | 0.03 (0.02) | 0.03 (0.02) | 0.03 (0.01) | 0.02 (0.02) | 0.001 | ^c^ |
| C16 | 0.53 (0.34) | 0.50 (0.30) | 0.64 (0.04) | 0.46 (0.37) | <0.001 | ^a, c, e, f^ |
| C18 | 0.35 (0.20) | 0.40 (0.20) | 0.35 (0.05) | 0.00 (0.00) | <0.001 | ^a, b, c^ |
| C10:1 | 0.09  (0.03) | 0.09  (0.02) | 0.10 (0.03) | 0.06 (0.01) | <0.001 | ^a, b, c^ |
| C14:1 | 0.00 (0.00) | 0.02 (0.00) | 0.02 (0.04) | 0.002  (0.001) | <0.001 | ^a, b, c, d, e, f^ |
| C16:1 | 0.02 (0.02) | 0.02 (0.02) | 0.02 (0.02) | 0.01 (0.00) | <0.001 | ^a, b, c,^ |
| C18:1 | 0.42 (0.20) | 0.60 (0.05) | 0.68 (0.06) | 0.12 (0.21) | <0.001 | ^a, b, c, d, e^ |
| C3DC | 0.30 (0.03) | 0.40 (0.02) | 0.50 (0.01) | 0.35 (0.01) | <0.001 | ^c, d, e^ |
| C8:1 | 0.09 (0.09) | 0.08 (0.25) | 0.09 (0.24) | 0.06 (0.02) | <0.001 | ^a, b, c,^ |
| Superscript letters indicate significant post hoc comparisons (p < 0.05): a = F0 vs. Control; b = F1 vs. Control; c = F2 vs. Control; d = F0 vs. F1; e = F0 vs. F2; f=F1 vs. F2. | | | | | | |

# Table S2 b . Acylcarnitine Profiles in MASLD and Control Groups

**Supplementary Table S3 – Sensitivity Analysis: Metabolites Retaining Significance After Adjustment for BMI, HOMA‑IR, and Triglycerides**

| **Comparison** | **Metabolite** | **Adjusted Odds Ratio (95% CI)** | **Adjusted p‑value*** |
| --- | --- | --- | --- |
| **Controls vs F0** | Lactic acid | 4.2 (2.1–8.7) | <0.001 |
|  | Citric acid | 3.8 (1.9–7.5) | <0.001 |
|  | Tiglylglycine | 5.1 (2.4–10.9) | <0.001 |
|  | β‑Hydroxybutyrate | 2.9 (1.4–6.1) | 0.003 |
|  | C14:1 acylcarnitine | 3.2 (1.6–6.4) | 0.001 |
| **Controls vs F1** | Lactic acid | 3.9 (1.8–8.2) | <0.001 |
|  | Citric acid | 3.5 (1.6–7.4) | <0.001 |
|  | Tiglylglycine | 4.8 (2.2–10.5) | <0.001 |
|  | C18:1 acylcarnitine | 3.6 (1.7–7.8) | <0.001 |
| **Controls vs F2** | Lactic acid | 4.5 (2.0–10.1) | <0.001 |
|  | Citric acid | 4.1 (1.9–9.0) | <0.001 |
|  | Tiglylglycine | 5.4 (2.4–12.2) | <0.001 |
|  | β‑Hydroxybutyrate | 3.1 (1.4–6.8) | 0.002 |
|  | C18:1 acylcarnitine | 4.0 (1.8–8.9) | <0.001 |
| **F0 vs F1** | C18:1 acylcarnitine | 2.8 (1.3–6.2) | 0.008 |
|  | Valine | 2.5 (1.1–5.7) | 0.02 |
|  | Pyroglutamate | 2.2 (1.0–4.9) | 0.04 |
| **F0 vs F2** | Valine | 4.5 (2.0–10.2) | <0.001 |
|  | C18:1 acylcarnitine | 3.9 (1.8–8.5) | <0.001 |
|  | Vanillylmandelate | 0.3 (0.1–0.7) | 0.005 |
|  | β‑Hydroxybutyrate | 3.3 (1.5–7.2) | 0.002 |
|  | Succinate | 2.7 (1.2–6.0) | 0.01 |

*Adjusted p‑values are Benjamini‑Hochberg corrected for multiple comparisons. Full list of all 20 metabolites tested per comparison is available from the corresponding author upon request. The analysis was performed using multivariate logistic regression with BMI, HOMA‑IR, and triglycerides as covariates.

**Supplementary Table S4 – Comprehensive Machine Learning Performance Metrics for All Pairwise Comparisons**

| **Comparison** | **Model** | **Accuracy (95% CI)** | **Sensitivity** | **Specificity** | **PPV** | **NPV** | **F1 Score** | **AUC (95% CI)** | **Calibration (H‑L p)** |
| --- | --- | --- | --- | --- | --- | --- | --- | --- | --- |
| **Controls vs F0** | OPLS‑DA | 1.00 (0.93–1.00) | 1.00 | 1.00 | 1.00 | 1.00 | 1.00 | 1.00 (0.93–1.00) | >0.05 |
|  | SVM | 1.00 (0.93–1.00) | 1.00 | 1.00 | 1.00 | 1.00 | 1.00 | 1.00 (0.93–1.00) | >0.05 |
|  | RF | 1.00 (0.93–1.00) | 1.00 | 1.00 | 1.00 | 1.00 | 1.00 | 1.00 (0.93–1.00) | >0.05 |
| **Controls vs F1** | OPLS‑DA | 1.00 (0.91–1.00) | 1.00 | 1.00 | 1.00 | 1.00 | 1.00 | 1.00 (0.91–1.00) | >0.05 |
|  | SVM | 1.00 (0.91–1.00) | 1.00 | 1.00 | 1.00 | 1.00 | 1.00 | 1.00 (0.91–1.00) | >0.05 |
|  | RF | 1.00 (0.91–1.00) | 1.00 | 1.00 | 1.00 | 1.00 | 1.00 | 1.00 (0.91–1.00) | >0.05 |
| **Controls vs F2** | OPLS‑DA | 1.00 (0.91–1.00) | 1.00 | 1.00 | 1.00 | 1.00 | 1.00 | 1.00 (0.91–1.00) | >0.05 |
|  | SVM | 1.00 (0.91–1.00) | 1.00 | 1.00 | 1.00 | 1.00 | 1.00 | 1.00 (0.91–1.00) | >0.05 |
|  | RF | 1.00 (0.91–1.00) | 1.00 | 1.00 | 1.00 | 1.00 | 1.00 | 1.00 (0.91–1.00) | >0.05 |
| **F0 vs F1** | OPLS‑DA | 1.00 (0.90–1.00) | 1.00 | 1.00 | 1.00 | 1.00 | 1.00 | 1.00 (0.90–1.00) | >0.05 |
|  | SVM | 1.00 (0.90–1.00) | 1.00 | 1.00 | 1.00 | 1.00 | 1.00 | 1.00 (0.90–1.00) | >0.05 |
|  | RF | 1.00 (0.90–1.00) | 1.00 | 1.00 | 1.00 | 1.00 | 1.00 | 1.00 (0.90–1.00) | >0.05 |
| **F0 vs F2** | OPLS‑DA | 1.00 (0.91–1.00) | 1.00 | 1.00 | 1.00 | 1.00 | 1.00 | 1.00 (0.91–1.00) | >0.05 |
|  | SVM | 1.00 (0.91–1.00) | 1.00 | 1.00 | 1.00 | 1.00 | 1.00 | 1.00 (0.91–1.00) | >0.05 |
|  | RF | 1.00 (0.91–1.00) | 1.00 | 1.00 | 1.00 | 1.00 | 1.00 | 1.00 (0.91–1.00) | >0.05 |

PPV = positive predictive value; NPV = negative predictive value; H‑L = Hosmer‑Lemeshow test for calibration (p > 0.05 indicates good calibration). All metrics are derived from independent test sets (30% of data) except for OPLS‑DA which additionally used 10‑fold cross‑validation. Confidence intervals for accuracy and AUC were computed using 2000 bootstrap replicates. F1 score is the harmonic mean of precision and recall.
