# Supplementary material for: Machine learning-based identification of targeted metabolomic biomarkers for early diagnosis and fibrosis-stage discrimination in metabolic dysfunction-associated steatotic liver disease
Source: Front Nutr. 2026 Aug 24;13:1849329. doi: 10.3389/fnut.2026.1849329 (PMC13547064; doi:10.3389/fnut.2026.1849329)
Supplement: Supplementary file 3 [file Table_3.DOCX]

**Supplementary Materials**

**Machine Learning-Based Identification of Targeted Metabolomic Biomarkers for Early Diagnosis and Fibrosis-Stage Discrimination in Metabolic Dysfunction-Associated Steatotic Liver Disease (MASLD)**

*Manuscript ID 1849329 • Original Research Article*

This document consolidates the machine-learning reproducibility materials for the three modelling architectures used in the study: orthogonal partial least squares discriminant analysis (OPLS-DA), random forest (RF), and support vector machine (SVM). All results were generated on the analysed cohort of 232 participants, 100 controls, and 132 patients with MASLD (68 F0, 34 F1, 30 F2). Full analysis scripts and per-comparison output files are provided as separate Supplementary Data/Code files and will be deposited in a public repository (GitHub with a Zenodo DOI; metabolomic data in MetaboLights) on acceptance.

**Contents**

• Supplementary Tables S1–S7 (variable ranges; metabolite profiles; sensitivity analysis; machine-learning performance and calibration; VIP rankings; file inventories; software environment)

• Supplementary Figures S1–S13 (correlation heatmaps; volcano plots; pathway enrichment; per-comparison model-validation panels)

• Supplementary Code S-Code 1–3 (OPLS-DA, random forest, SVM)

**Reproducibility overview and software environment**

All analyses were performed in R (v4.6.0; x86_64-w64-mingw32, Windows 11 x64). Random seeds were fixed in every script (OPLS-DA: set. seed(42); RF: set. seed(1234) with per-repeat offsets; permutation nulls seeded independently) so that every reported value is exactly reproducible. The complete sessionInfo() output (attached packages and versions) is provided as a Supplementary Data file (sessionInfo).

**Key package versions.**

| **Package** | **Version** | **Role in pipeline** |
| --- | --- | --- |
| ropls | 1.44.0 | OPLS-DA model fitting, VIP scores, permutation testing |
| randomForest | 4.7-1.2 | Random forest classifier |
| ranger | 0.18.0 | Fast RF backend/importance |
| e1071 | 1.7-17 | Support vector machine (RBF kernel) |
| caret | 7.0-1 | Cross-validation, pre-processing, tuning, and confusion matrices |
| Boruta | 10.0.0 | All-relevant feature selection (within training folds) |
| pROC | 1.19.0.1 | ROC/AUC estimation |
| tidymodels / rsample | 1.5.0 / 1.3.2 | Resampling and stratified splits |
| writexl / openxlsx | 1.5.4 / 4.2.8.1 | Summary workbook export |

*Table S-env. Principal R packages and versions (full environment in the sessionInfo Supplementary Data file).*

**Complete inventory of analysis, code, and output files**

For full transparency, all files generated by the three modelling pipelines are listed below. These files are provided as Supplementary Data and reproduce all figures, tables, and statistics in the manuscript from the raw metabolite matrix.

**S2.1 OPLS-DA pipeline (OPLSDA_Results/)**

| **File/folder** | **Contents** |
| --- | --- |
| OPLSDA_script.txt | Full annotated R script (S-Code 1) |
| OPLSDA_Statistical_Summary.xlsx | Master workbook: model statistics + per-comparison VIP sheets |
| Validation_Summary_AllComparisons.csv | R²Y, Q², permutation p-values and Valid_Model flag, all comparisons |
| VIP_Tables/ | Ranked VIP-score CSV per comparison (5 files) |
| VIP_Plots/ | VIP bar chart per comparison, 300 dpi TIFF (5 files) |
| Score_Plots/ | OPLS-DA score plot (t1 vs to1) per comparison (5 files) |
| Permutation_Plots/ | Permutation-diagnostic plot per comparison (5 files) |
| Permutation_Raw_Data/ | Full 1,000-permutation R²X/R²Y/Q² matrix per comparison (5 files) |
| Outlier_Diagnostics/ | Score-distance vs orthogonal-distance outlier plot per comparison (5 files) |
| Summary_Panels/ | Combined 4-panel diagnostic figure per comparison (5 files) |
| Boruta_Feature_Selection/ | Boruta all-relevant selection output (see §S6) |
| OPLSDA_Reviewer_Proof_Results.zip | Archived copy of the complete OPLS-DA output tree |

*Table S2.1. OPLS-DA output inventory. permI = 1,000 permutations per comparison.*

**S2.2 Random-forest pipeline (RF_Reviewer_Proof_Results/)**

| **File/folder** | **Contents** |
| --- | --- |
| Code script for Random Forest.txt | Full annotated R script leak-free repeated nested CV (S-Code 2) |
| data_audit Ra.txt | Structure/class/NA audit of the raw matrix (232 obs × 51 variables) |
| Model_Performance_Summary.xlsx | Nested-CV AUC/Accuracy/Sensitivity/Specificity/F1/MCC (Table S2) |
| Permutation_Test_Summary.xlsx | Empirical null (label-shuffle) AUC distribution per comparison |
| Feature_Selection_Frequency.xlsx | Boruta selection frequency across all outer folds/repeats |
| Fold_Splits_Audit.csv | Train/test IDs for every outer fold and repeat (leakage audit) |
| Imputation_Audit.csv | Log proving test-fold imputation used TRAIN-derived medians only |
| RF_Importance_*.xlsx / RF_Importance2_*.xlsx | Per-comparison variable-importance tables (10 files) |
| Separation_Diagnostic.xlsx / _Round2.xlsx | Class-separation diagnostics |
| Importance_Plots/ , Saved_Models/ | Importance figures and serialised fitted models (.rds) |
| final_results_full.rds | Complete R results object for exact reproduction |
| *_NoArtifacts.* (parallel set) | Sensitivity re-run after excluding technical-artifact features |
| sessionInfo.txt / sessionInfo2.txt | Software environment |

*Table S2.2. Random forest output inventory. The parallel ’ NoArtifacts’ files repeat the entire pipeline with technical-artefact features removed, confirming the results are not artefact-driven.*

**Support vector machine pipeline (SVM_Reviewer_Proof_Results/)**

| **File/folder** | **Contents** |
| --- | --- |
| SVM analysis script (S-Code 3) | Full annotated R script to be provided with the SVM output tree |
| Model_Performance_Summary.xlsx | Nested-CV AUC/Accuracy/Sensitivity/Specificity/F1/MCC (Table S3) |
| Permutation_Test_Summary.xlsx | Empirical null (label-shuffle) AUC distribution per comparison |
| Feature_Selection_Frequency.xlsx | Boruta selection frequency across folds |
| Fold_Splits_Audit.csv | Train/test IDs for every fold (leakage audit) |
| Imputation_Audit.csv | Train-derived imputation log |
| SVM_Importance_*.xlsx | Per-comparison coefficient-magnitude importance tables (5 files) |
| Importance_Plots/ , Saved_Models/ | Importance figures and serialised models |
| final_results_svm_full.rds | Complete R results object |
| sessionInfo SVM.txt | Software environment |

*Table S2.3. Support vector machine output inventory (radial-basis-function kernel).*

Available at the link:

<https://texastechuniversity-my.sharepoint.com/personal/mosabdel_ttu_edu/_layouts/15/onedrive.aspx?id=%2Fpersonal%2Fmosabdel_ttu_edu%2FDocuments%2FDr.%20Shimma&ga=1>

Other Link:

<https://drive.google.com/drive/folders/1Mdk25vBqrqBSa4jHtrq-WRT-JD4b2tMu>

**1. Supplementary Tables**

**Table S1.** Complete ranges of the measured variables (minimum–maximum per group: controls, F0, F1, F2).

| Variable | F0 (n=68) | F1 (n=34) | F2 (n=30) | Controls (n=100) |
| --- | --- | --- | --- | --- |
| Glucose (mg/dL) | 76 – 99 | 79 – 97 | 80 – 97 | 73 – 91 |
| BMI (kg/m²) | 27.1 – 33.0 | 29.6 – 33.8 | 30.2 – 34.0 | 20.6 – 22.4 |
| Waist circumference (cm) | 97.9 – 110.9 | 95.2 – 110.1 | 98.9 – 111.5 | 66.2 – 71.9 |
| Hip circumference (cm) | 108.5 – 116.2 | 100.7 – 122.0 | 108.0 – 129.4 | 84.8 – 94.4 |
| Waist‑to‑hip ratio | 0.88 – 0.98 | 0.86 – 1.00 | 0.83 – 0.98 | 0.74 – 0.80 |
| Systolic BP (mmHg) | 110 – 135 | 110 – 140 | 110 – 140 | 100 – 125 |
| Diastolic BP (mmHg) | 70 – 90 | 70 – 100 | 80 – 90 | 70 – 80 |

*Ranges are the minimum–maximum values observed in each group.*

**Table S2.** Plasma amino-acid, acylcarnitine and organic-acid profiles among MASLD stages and controls, with raw and FDR-adjusted p-values. Part (a): amino acids and acylcarnitines; part (b): organic acids.

| Variable | F0  N=68 | F1  N=34 | F2  N=30 | Controls  N=100 | p-value | Post-hoc test |
| --- | --- | --- | --- | --- | --- | --- |
| Glutaric | 3.73 (1.85) | 3.59 (2.22) | 3.58 (2.31) | 3.645 (1.88) | 0.3 | NA |
| Adipic | 3.79 (1.21) | 3.75 (1.71) | 3.595 (1.07) | 2.19 (0.94) | <0.001 | ^a, b, c^ |
| Pimelic | 1.63 (0.47) | 1.64 (0.47) | 1.655 (0.82) | 1.63 (0.11) | 0.83 | NA |
| Suberic | 1.59 (1.04) | 1.65 (1.09) | 1.875 (0.70) | 1.57 (0.18) | <0.001 | ^a, b, c^ |
| Sebacic | 4.42 (4.11) | 4.8 (4.12) | 1.63 (3.12) | 0.92 (0.00) | <0.001 | ^a, b, c^ |
| Methylmalonic | 4.69 (3.31) | 5.52 (2.86) | 4.575 (3.71) | 3.8 (1.59) | <0.001 | ^a, b, c^ |
| Ethylmalonate | 2.82 (1.84) | 1.59 (0.84) | 1.555 (1.08) | 3.76 (1.18) | <0.001 | ^a, b, c, d, e^ |
| Succinic | 4.4 (1.95) | 3.0 (1.00) | 3.4 (1.10) | 4.545 (1.48) | <0.001 | ^b, c, d, e^ |
| Citric Acid | 13.5 (2.42) | 13.8 (3.00) | 14.0 (3.30) | 3.57 (2.40) | <0.001 | ^a, b, c^ |
| Hydroxypropionic | 7.2 (2.00) | 6.4 (1.95) | 6.45 (1.70) | 3.565 (2.98) | <0.001 | ^a, b, c^ |
| Tiglylglycine | 3.92 (1.00) | 3.92 (1.90) | 3.92 (1.90) | 0.795 (0.29) | <0.001 | ^a, b, c^ |
| Lactic | 23.5 (6.45) | 23.5 (9.90) | 23.0 (2.40) | 3.165 (1.17) | <0.001 | ^a, b, c^ |
| ?-hydroxybutyrate | 7.1 (1.10) | 5.27 (0.69) | 4.375 (1.02) | 2.555 (0.55) | <0.001 | ^a, b, c, e^ |
| Vanillylmandelate | 2.1 (0.30) | 1.5 (0.35) | 1.4 (0.40) | 2.555 (0.63) | <0.001 | ^a, b, c, d, e^ |
| Pyroglutamate | 19.0 (3.00) | 17.0 (3.50) | 17.0 (3.00) | 24.205 (2.99) | <0.001 | ^a, b, c^ |
| p-hydroxyphenyllactate | 0.25 (0.11) | 0.24 (0.10) | 0.315 (0.18) | 0.21 (0.10) | <0.001 | ^a, b, c^ |
| Formiminoglutamate | 0.19 (0.12) | 0.17 (0.10) | 0.185 (0.09) | 0.65 (0.48) | <0.001 | ^a, b, c^ |
| ASP (ng/mL) | 180.33 (109.78) | 180.00 (29.50) | 182.93 (61.95) | 49.00 (18.70) | <0.00 | ^a, b, c^ |
| CIT:Phe | 0.38 (0.16) | 0.44 (0.17) | 0.25 (0.05) | 0.33 (0.53) | <0.00 | ^a, b, c, f^ |
| Citrulline | 27.50 (5.70) | 28.80 (4.82) | 21.34 (2.30) | 19.20 (9.82) | <0.00 | ^a, b, e, f^ |
| Glutamine | 130.50 (45.45) | 117.50 (23.06) | 195.25 (65.01) | 93.50 (41.02) | <0.00 | ^a, b, c, e, f^ |
| Glycine | 175.00 (48.24) | 155.50 (17.08) | 150.28 (14.44) | 207.00 (54.25) | <0.00 | ^a, b, c, d^ |
| Leu/Ile | 124.00 (23.10) | 134.50 (8.28) | 135.85 (7.55) | 82.45 (20.00) | <0.00 | ^a, b, c, e^ |
| Methionine | 24.17 (2.85) | 24.96 (1.64) | 24.98 (2.74) | 25.00 (2.00) | 0.05 | ^NA^ |
| Ornithine | 80.21 (16.00) | 80.45 (19.00) | 87.47 (33.00) | 100.00 (16.00) | <0.00 | ^a, b, c^ |
| Proline | 104.40 (36.20) | 110.18 (35.10) | 95.35 (15.03) | 90.40 (30.52) | <0.00 | ^a, b^ |
| Phenylalanine | 54.14 (6.47) | 57.00 (4.95) | 54.69 (3.42) | 54.00 (3.00) | <0.00 | ^b^ |
| Tyrosine | 50.00 (9.38) | 50.70 (6.17) | 58.79 (4.26) | 48.00 (5.38) | <0.00 | ^a, b, c, e, f^ |
| Valine | 75.50 (6.88) | 84.00 (5.18) | 86.15 (3.87) | 66.17 (16.96) | <0.00 | ^a, b, c, d, e^ |
| Alanine | 305.30 (32.94) | 320.19 (28.73) | 347.35 (27.80) | 290.00 (53.50) | <0.00 | ^b, c, d, e^ |
| Arginine | 8.36 (2.50) | 11.32 (2.71) | 9.44 (3.27) | 6.00 (3.92) | <0.00 | ^a, b, c, d^ |
| Superscript letters indicate significant post hoc comparisons (p < 0.05): a = F0 vs. Control; b = F1 vs. Control; c = F2 vs. Control; d = F0 vs. F1; e = F0 vs. F2; f=F1 vs. F2. | | | | | | |

**Table S3.** Sensitivity analysis for metabolic confounding odds ratios adjusted for BMI, HOMA-IR and triglycerides (multivariable logistic regression; Benjamini–Hochberg-corrected).

| **Comparison** | **Metabolite** | **Adjusted OR** | **95% CI** | **p-value** |
| --- | --- | --- | --- | --- |
| Controls vs F0 | Lactic acid | 4.2 | 2.1–8.7 | <0.001 |
| Controls vs F0 | Citric acid | 3.8 | 1.9–7.5 | <0.001 |
| Controls vs F0 | Tiglylglycine | 5.1 | 2.4–10.9 | <0.001 |
| Controls vs F0 | β-hydroxybutyrate | 2.9 | 1.4–6.1 | 0.003 |
| Controls vs F0 | C14:1 | 3.2 | 1.6–6.4 | 0.001 |
| F0 vs F2 | Valine | 4.5 | 2.0–10.2 | <0.001 |
| F0 vs F2 | C18:1 | 3.9 | 1.8–8.5 | <0.001 |
| F0 vs F2 | Vanillylmandelate | 0.3 | 0.1–0.7 | 0.005 |

*Only metabolites remaining significant after adjustment are shown; complete model output is provided in the Supplementary Data. An adjusted OR < 1 (vanillylmandelate) indicates lower odds with advancing fibrosis.*

**Table S4. Comprehensive machine-learning performance and calibration.**

These repeated nested cross-validation results are the robustness analysis referenced in the main text; they complement the independent held-out test-set performance in Table 3 and use the same leakage-free pipeline (pre-processing and feature selection re-derived within training folds only).

**Table S4a.** OPLS-DA model statistics (1 predictive + 1 orthogonal component; standard scaling). Permutation p-values are for R²Y and Q² over 1,000 permutations; values are the authoritative outputs of the final 232-participant analysis.

| **Comparison** | **n (grp1/grp2)** | **N** | **R²X(cum)** | **R²Y(cum)** | **Q²(cum)** | **p(R²Y)** | **p(Q²)** | **Valid** |
| --- | --- | --- | --- | --- | --- | --- | --- | --- |
| F0 vs Control | 68 / 100 | 168 | 0.364 | 0.976 | 0.971 | 0.001 | 0.001 | Yes |
| F1 vs Control | 34 / 100 | 134 | 0.407 | 0.983 | 0.978 | 0.001 | 0.001 | Yes |
| F2 vs Control | 30 / 100 | 130 | 0.414 | 0.976 | 0.971 | 0.001 | 0.001 | Yes |
| F1 vs F0 | 34 / 68 | 102 | 0.151 | 0.828 | 0.720 | 0.001 | 0.001 | Yes |
| F2 vs F0 | 30 / 68 | 98 | 0.201 | 0.928 | 0.888 | 0.001 | 0.001 | Yes |

**Table S4b.** Random-forest repeated nested-CV performance (mean ± SD; 5 outer folds × 10 repeats = 50 held-out evaluations; inner 5-fold tuning; Boruta selection re-derived inside every training fold). MCC = Matthews correlation coefficient.

| **Comparison** | **AUC** | **Accuracy** | **Sensitivity** | **Specificity** | **F1** | **MCC** |
| --- | --- | --- | --- | --- | --- | --- |
| F0 vs Control | 1.000 ± 0.000 | 1.000 ± 0.000 | 1.000 ± 0.000 | 1.000 ± 0.000 | 1.000 ± 0.000 | 1.000 ± 0.000 |
| F1 vs Control | 1.000 ± 0.000 | 1.000 ± 0.000 | 1.000 ± 0.000 | 1.000 ± 0.000 | 1.000 ± 0.000 | 1.000 ± 0.000 |
| F2 vs Control | 1.000 ± 0.000 | 1.000 ± 0.000 | 1.000 ± 0.000 | 1.000 ± 0.000 | 1.000 ± 0.000 | 1.000 ± 0.000 |
| F1 vs F0 | 1.000 ± 0.000 | 0.988 ± 0.032 | 0.965 ± 0.097 | 1.000 ± 0.000 | 0.979 ± 0.057 | 0.974 ± 0.071 |
| F2 vs F0 | 1.000 ± 0.000 | 1.000 ± 0.000 | 1.000 ± 0.000 | 1.000 ± 0.000 | 1.000 ± 0.000 | 1.000 ± 0.000 |

*The metabolically matched F1 vs F0 contrast does not reach perfection (MCC 0.974 ± 0.071), the expected behaviour for a genuinely harder, adjacent-stage comparison.*

**Table S4c.** Support-vector-machine repeated nested-CV performance (mean ± SD; 50 outer evaluations; radial-basis-function kernel) under the identical framework.

| **Comparison** | **AUC** | **Accuracy** | **Sensitivity** | **Specificity** | **F1** | **MCC** |
| --- | --- | --- | --- | --- | --- | --- |
| F0 vs Control | 1.000 ± 0.000 | 0.993 ± 0.016 | 1.000 ± 0.000 | 0.988 ± 0.028 | 0.992 ± 0.019 | 0.986 ± 0.032 |
| F1 vs Control | 1.000 ± 0.000 | 0.999 ± 0.010 | 0.994 ± 0.040 | 1.000 ± 0.000 | 0.997 ± 0.024 | 0.996 ± 0.027 |
| F2 vs Control | 1.000 ± 0.000 | 0.998 ± 0.008 | 1.000 ± 0.000 | 0.998 ± 0.010 | 0.997 ± 0.015 | 0.996 ± 0.019 |
| F1 vs F0 | 0.994 ± 0.015 | 0.967 ± 0.048 | 0.963 ± 0.086 | 0.969 ± 0.052 | 0.951 ± 0.071 | 0.930 ± 0.104 |
| F2 vs F0 | 1.000 ± 0.000 | 1.000 ± 0.000 | 1.000 ± 0.000 | 1.000 ± 0.000 | 1.000 ± 0.000 | 1.000 ± 0.000 |

*As with RF, the adjacent-stage F1 vs F0 comparison is the hardest (MCC 0.930 ± 0.104; AUC 0.994 ± 0.015), whereas case-detection contrasts approach ceiling a gradient that tracks genuine biological difficulty rather than leakage.*

**Model calibration was adequate for five comparisons (Hosmer–Lemeshow goodness-of-fit p > 0.05).**

**Table S5. Ranked VIP tables (all comparisons).**

Top-20 metabolites by variable-importance-in-projection (VIP) score for each OPLS-DA comparison (full 51-metabolite tables in the VIP_Tables/ folder). VIP > 1 denotes an above-average contribution to class separation.

**Table S5a.** VIP ranking F0 vs Control

| **Rank** | **Metabolite** | **VIP** | **Rank** | **Metabolite** | **VIP** |
| --- | --- | --- | --- | --- | --- |
| 1 | Lactic | 1.786 | 11 | Hydroxy-propionic | 1.235 |
| 2 | Citric acid | 1.756 | 12 | C10:1 | 1.211 |
| 3 | Tiglylglycine | 1.754 | 13 | Adipic | 1.211 |
| 4 | β-hydroxybutyrate | 1.726 | 14 | C3 | 1.187 |
| 5 | C14:1 | 1.670 | 15 | C0 | 1.165 |
| 6 | ASP (aspartate) | 1.627 | 16 | Pyroglutamate | 1.147 |
| 7 | C18:1 | 1.470 | 17 | C2 | 1.143 |
| 8 | Leu/Ile | 1.404 | 18 | Valine | 1.057 |
| 9 | Formiminoglutamate | 1.397 | 19 | C10 | 1.021 |
| 10 | Sebacic | 1.385 | 20 | C5-DC | 1.013 |

**Table S5b.** VIP ranking — F1 vs Control

| **Rank** | **Metabolite** | **VIP** | **Rank** | **Metabolite** | **VIP** |
| --- | --- | --- | --- | --- | --- |
| 1 | C14:1 | 1.706 | 11 | C10:1 | 1.348 |
| 2 | Lactic | 1.692 | 12 | C0 | 1.325 |
| 3 | ASP (aspartate) | 1.650 | 13 | Ethylmalonate | 1.217 |
| 4 | Tiglylglycine | 1.641 | 14 | Valine | 1.203 |
| 5 | Citric acid | 1.630 | 15 | Formiminoglutamate | 1.170 |
| 6 | C18:1 | 1.608 | 16 | C2 | 1.169 |
| 7 | β-hydroxybutyrate | 1.579 | 17 | Pyroglutamate | 1.145 |
| 8 | Leu/Ile | 1.426 | 18 | Adipic | 1.134 |
| 9 | C3 | 1.390 | 19 | Glycine | 1.130 |
| 10 | Sebacic | 1.351 | 20 | Arg | 1.127 |

**Table S5c.** VIP ranking — F2 vs Control

| **Rank** | **Metabolite** | **VIP** | **Rank** | **Metabolite** | **VIP** |
| --- | --- | --- | --- | --- | --- |
| 1 | Lactic | 1.676 | 11 | Valine | 1.206 |
| 2 | Tiglylglycine | 1.639 | 12 | C10:1 | 1.183 |
| 3 | Citric acid | 1.624 | 13 | Ethylmalonate | 1.179 |
| 4 | C18:1 | 1.599 | 14 | Adipic | 1.178 |
| 5 | ASP (aspartate) | 1.577 | 15 | Formiminoglutamate | 1.136 |
| 6 | C0 | 1.482 | 16 | Glycine | 1.116 |
| 7 | β-hydroxybutyrate | 1.457 | 17 | C2 | 1.105 |
| 8 | C14:1 | 1.410 | 18 | C4 | 1.103 |
| 9 | Leu/Ile | 1.376 | 19 | Glutamine | 1.083 |
| 10 | C3 | 1.345 | 20 | Pyroglutamate | 1.080 |

**Table S5d.** VIP ranking — F1 vs F0

| **Rank** | **Metabolite** | **VIP** | **Rank** | **Metabolite** | **VIP** |
| --- | --- | --- | --- | --- | --- |
| 1 | C18:1 | 2.508 | 11 | Leu/Ile | 1.437 |
| 2 | Valine | 2.418 | 12 | C10 | 1.071 |
| 3 | Succinic | 2.237 | 13 | Ethylmalonate | 1.013 |
| 4 | Arg | 1.945 | 14 | C16 | 0.909 |
| 5 | β-hydroxybutyrate | 1.808 | 15 | C16:1 | 0.879 |
| 6 | C5 | 1.671 | 16 | C10:1 | 0.869 |
| 7 | Alanine | 1.616 | 17 | C2 | 0.810 |
| 8 | C18 | 1.581 | 18 | Glutamine | 0.800 |
| 9 | Glycine | 1.518 | 19 | C8 | 0.788 |
| 10 | Pyroglutamate | 1.440 | 20 | Methionine | 0.718 |

**Table S5e.** VIP ranking — F2 vs F0

| **Rank** | **Metabolite** | **VIP** | **Rank** | **Metabolite** | **VIP** |
| --- | --- | --- | --- | --- | --- |
| 1 | Vanillylmandelate | 2.371 | 11 | Glycine | 1.277 |
| 2 | Valine | 2.168 | 12 | Citrulline | 1.268 |
| 3 | β-hydroxybutyrate | 2.083 | 13 | Succinic | 1.150 |
| 4 | C18:1 | 2.065 | 14 | C3-DC | 1.016 |
| 5 | C14:1 | 1.756 | 15 | Arg | 0.994 |
| 6 | CIT/Phe | 1.748 | 16 | Proline | 0.932 |
| 7 | Alanine | 1.644 | 17 | C5-DC | 0.911 |
| 8 | Tyrosine | 1.578 | 18 | C10 | 0.910 |
| 9 | Glutamine | 1.463 | 19 | C4 | 0.883 |
| 10 | C5 | 1.290 | 20 | Leu/Ile | 0.805 |

**Table S6. Analysis, code and output-file inventories (three pipelines).**

For full transparency, all files generated by the three modelling pipelines are listed below. These reproduce every figure, table and statistic in the manuscript from the raw metabolite matrix.

**Table S6a.** OPLS-DA output inventory (permI = 1,000 permutations per comparison).

| **File/folder** | **Contents** |
| --- | --- |
| OPLSDA_script.txt | Full annotated R script (S-Code 1) |
| OPLSDA_Statistical_Summary.xlsx | Master workbook: model statistics + per-comparison VIP sheets |
| Validation_Summary_AllComparisons.csv | R²Y, Q², permutation p-values and Valid_Model flag, all comparisons |
| VIP_Tables/ | Ranked VIP-score CSV per comparison (5 files) |
| VIP_Plots/ | VIP bar chart per comparison, 300 dpi TIFF (5 files) |
| Score_Plots/ | OPLS-DA score plot (t1 vs to1) per comparison (5 files) |
| Permutation_Plots/ | Permutation-diagnostic plot per comparison (5 files) |
| Permutation_Raw_Data/ | Full 1,000-permutation R²X/R²Y/Q² matrix per comparison (5 files) |
| Outlier_Diagnostics/ | Score-distance vs orthogonal-distance outlier plot per comparison (5 files) |
| Summary_Panels/ | Combined 4-panel diagnostic figure per comparison (5 files) |
| Boruta_Feature_Selection/ | Boruta all-relevant selection output (see §S6) |
| OPLSDA_Reviewer_Proof_Results.zip | Archived copy of the complete OPLS-DA output tree |

**Table S6b.** Random-forest output inventory. The parallel '…NoArtifacts' files repeat the pipeline with technical-artefact features removed, confirming the results are not artefact-driven.

| **File/folder** | **Contents** |
| --- | --- |
| Code script for Random Forest.txt | Full annotated R script — leak-free repeated nested CV (S-Code 2) |
| data_audit Ra.txt | Structure/class/NA audit of the raw matrix (232 obs × 51 variables) |
| Model_Performance_Summary.xlsx | Nested-CV AUC/Accuracy/Sensitivity/Specificity/F1/MCC (Table S2) |
| Permutation_Test_Summary.xlsx | Empirical null (label-shuffle) AUC distribution per comparison |
| Feature_Selection_Frequency.xlsx | Boruta selection frequency across all outer folds/repeats |
| Fold_Splits_Audit.csv | Train/test IDs for every outer fold and repeat (leakage audit) |
| Imputation_Audit.csv | Log proving test-fold imputation used TRAIN-derived medians only |
| RF_Importance_*.xlsx / RF_Importance2_*.xlsx | Per-comparison variable-importance tables (10 files) |
| Separation_Diagnostic.xlsx / _Round2.xlsx | Class-separation diagnostics |
| Importance_Plots/ , Saved_Models/ | Importance figures and serialised fitted models (.rds) |
| final_results_full.rds | Complete R results object for exact reproduction |
| *_NoArtifacts.* (parallel set) | Sensitivity re-run after excluding technical-artifact features |
| sessionInfo.txt / sessionInfo2.txt | Software environment |

**Table S6c.** Support-vector-machine output inventory (radial-basis-function kernel).

| **File/folder** | **Contents** |
| --- | --- |
| SVM analysis script (S-Code 3) | Full annotated R script — to be provided with the SVM output tree |
| Model_Performance_Summary.xlsx | Nested-CV AUC/Accuracy/Sensitivity/Specificity/F1/MCC (Table S3) |
| Permutation_Test_Summary.xlsx | Empirical null (label-shuffle) AUC distribution per comparison |
| Feature_Selection_Frequency.xlsx | Boruta selection frequency across folds |
| Fold_Splits_Audit.csv | Train/test IDs for every fold (leakage audit) |
| Imputation_Audit.csv | Train-derived imputation log |
| SVM_Importance_*.xlsx | Per-comparison coefficient-magnitude importance tables (5 files) |
| Importance_Plots/ , Saved_Models/ | Importance figures and serialised models |
| final_results_svm_full.rds | Complete R results object |
| sessionInfo SVM.txt | Software environment |

Supplementary Data repository (SharePoint):

https://texastechuniversity-my.sharepoint.com/personal/mosabdel_ttu_edu/_layouts/15/onedrive.aspx?id=%2Fpersonal%2Fmosabdel_ttu_edu%2FDocuments%2FDr.%20Shimma&ga=1

Mirror (Google Drive): https://drive.google.com/drive/folders/1Mdk25vBqrqBSa4jHtrq-WRT-JD4b2tMu

**Software environment**

All analyses were performed in R (v4.6.0; x86_64-w64-mingw32, Windows 11 x64). Random seeds were fixed in every script (OPLS-DA set. seed(42); RF set. seed(1234) with per-repeat offsets; permutation nulls seeded independently) so that every reported value is exactly reproducible. The complete sessionInfo() output is provided as a Supplementary Data file.

**Table S7.** Principal R packages (full environment in the sessionInfo Supplementary Data file).

| **Package** | **Version** | **Role in pipeline** |
| --- | --- | --- |
| ropls | 1.44.0 | OPLS-DA model fitting, VIP scores, permutation testing |
| randomForest | 4.7-1.2 | Random forest classifier |
| ranger | 0.18.0 | Fast RF backend/importance |
| e1071 | 1.7-17 | Support vector machine (RBF kernel) |
| caret | 7.0-1 | Cross-validation, pre-processing, tuning, and confusion matrices |
| Boruta | 10.0.0 | All-relevant feature selection (within training folds) |
| pROC | 1.19.0.1 | ROC/AUC estimation |
| tidymodels / rsample | 1.5.0 / 1.3.2 | Resampling and stratified splits |
| writexl / openxlsx | 1.5.4 / 4.2.8.1 | Summary workbook export |

**2. Reproducibility notes**

Feature selection (Boruta). To provide a feature-selection method independent of standard VIP ranking, the RF and SVM pipelines used the Boruta all-relevant wrapper (maxRuns = 150), applied only within each training fold. Boruta compares each real feature against randomised 'shadow' features and retains only those confirmed to carry more information than the best shadow feature. Because selection is re-executed in every outer fold and repeat, the stability of each feature is quantified by its selection frequency across the 50 folds. Features confirmed by Boruta in the large majority of folds coincide with the top-ranked VIP metabolites (lactic acid, citric acid, tiglylglycine, β-hydroxybutyrate, C14:1, C18:1, valine, vanillylmandelate), giving convergent, VIP-independent support for the reported panels.

*Nomenclature note: the algorithm used is Boruta (an all-relevant random-forest wrapper); this is distinct from a Borda count (a rank-aggregation scheme).*

Supplementary code. The complete, executable R scripts are provided as plain-text Supplementary Code files so the entire pipeline can be inspected and re-run line by line:

**• S-Code 1. OPLSDA_script.txt** — OPLS-DA fitting, VIP extraction, 1,000-permutation testing and all diagnostic outputs.

**• S-Code 2. Code script for Random Forest.txt** — leak-free repeated nested cross-validation (5×10), Boruta feature selection within training folds, inner 5-fold tuning, and a 100-permutation empirical null.

**• S-Code 3. SVM analysis script** — the parallel SVM pipeline (radial-basis-function kernel) under the identical leak-free framework.

Each script begins with a header documenting its purpose, the random seed, the required packages, and the exact leakage-prevention protocol (all pre-processing, imputation and feature selection are fitted on the training data only and then applied unchanged to the held-out data).

**3. Supplementary Figures**

The panels below are the original-resolution multivariate, univariate, pathway-enrichment and model-validation figures supporting the main text.

**Supplementary Figure S1.** Metabolite–metabolite and group correlation heatmaps (Pearson correlation) with unsupervised clustering, by domain: (A) acylcarnitines; (B) clinical/biochemical measures; (C) amino acids; (D) organic acids; (E) non-invasive fibrosis scores and imaging metrics; (F) anthropometric and haemodynamic variables.

**Supplementary Figure S2.** Volcano plot, controls vs F0. log₂ fold-change versus –log₁₀(FDR).

*Volcano plot of the univariate analysis distinguishing controls from F0. x-axis, log₂ fold change; y-axis, –log₁₀(FDR). Coloured points denote significantly up- or down-regulated metabolites; grey points are non-significant. Horizontal dashed line, FDR 0.05; vertical dashed lines, fold change |1.5|.*

Volcano plot, controls vs F0. log₂ fold-change versus –log₁₀ p-value. Lactic acid, citric acid, tiglylglycine, β-hydroxybutyrate, C14:1, and C18:1 are the most strongly up-regulated organic acids and acylcarnitines.

**Supplementary Figure S3.** Volcano plot, controls vs F1 (mild fibrosis). log₂ fold-change versus –log₁₀(FDR).

**Supplementary Figure S4.** Volcano plot, controls vs F2 (moderate fibrosis). log₂ fold-change versus –log₁₀(FDR).Volcano plot, controls vs F2 (moderate fibrosis). log₂ fold-change versus –log₁₀ p-value.

**Supplementary Figure S5.** Volcano plot, F0 vs F1 (stage stratification). log₂ fold-change versus –log₁₀(FDR).Volcano plot, F0 vs F1 (stage stratification). log₂ fold-change versus –log₁₀ p-value.

**Supplementary Figure S6.** Volcano plot, F0 vs F2 (stage stratification). log₂ fold-change versus –log₁₀(FDR).Volcano plot, F0 vs F2 (stage stratification). log₂ fold-change versus –log₁₀ p-value.

**Supplementary Figure S7.** Pathway-enrichment overview, case-discrimination panel. Metabolite sets ranked by –log₁₀ p-value; point size proportional to enrichment ratio.

*Enrichment of metabolic pathways for the case-discrimination panel (Panel 1).*

*v*Pathway-enrichment overview, case discrimination. Top enriched metabolite sets ranked by –log₁₀ p-value; point size denotes enrichment ratio, and colour denotes p-value

**Supplementary Figure S8.** Pathway-enrichment overview, stage-stratification panel.

*Enrichment of metabolic pathways for the stage-stratification panel (Panel 2).*

Pathway-enrichment overview, stage stratification. Top enriched metabolite sets ranked by –log₁₀ p-value; point size denotes enrichment ratio, and colour denotes p-value.

**Supplementary Figure S9.** Model-validation summary, controls vs F0: permutation tests (accuracy and R²Y/Q²), ROC curves and predicted-probability separation for OPLS-DA, RF and SVM.

*Model-validation summary: permutation tests (accuracy and R²Y/Q²), ROC curve, and predicted-probability distributions. The observed performance lies far outside the permutation null (OPLS-DA permutation p ≤ 0.001 over 1,000 permutations; random-forest empirical p ≈ < 0.01 over 100 permutations), evidence against data leakage.*

**Supplementary Figure S10.** Model-validation summary, controls vs F1 (panels as in Supplementary Figure S9).

*Model-validation summary: permutation tests (accuracy and R²Y/Q²), ROC curve, and predicted-probability distributions. The observed performance lies far outside the permutation null (OPLS-DA permutation p ≤ 0.001 over 1,000 permutations; random-forest empirical p ≈ < 0.01 over 100 permutations), evidence against data leakage.*

**Supplementary Figure S11.** Model-validation summary, controls vs F2 (panels as in Supplementary Figure S9).

*Model-validation summary: permutation tests (accuracy and R²Y/Q²), ROC curve, and predicted-probability distributions. The observed performance lies far outside the permutation null (OPLS-DA permutation p ≤ 0.001 over 1,000 permutations; random-forest empirical p ≈ < 0.01 over 100 permutations), evidence against data leakage.*

**Supplementary Figure S12.** Model-validation summary, F0 vs F1 (panels as in Supplementary Figure S9).

*Model-validation summary: permutation tests (accuracy and R²Y/Q²), ROC curve, and predicted-probability distributions. The observed performance lies far outside the permutation null (OPLS-DA permutation p ≤ 0.001 over 1,000 permutations; random-forest empirical p ≈ < 0.01 over 100 permutations), evidence against data leakage.*

**Supplementary Figure S13.** Model-validation summary, F0 vs F2 (panels as in Supplementary Figure S9).

**Metabolic differences between controls and F0**

**Supplementary Figure S14:** *Metabolic differences between controls and F0 using OPLS-DA, RF, and SVM. (A) Observation diagnostics; (B) OPLS-DA score plot; (C) top contributing metabolites by VIP score (OPLS-DA), mean decrease in Gini (RF) and SVM coefficients; (D) violin plots of selected metabolites (lactic acid, citric acid, tiglylglycine, β-hydroxybutyrate). Lactic acid, citric acid, and tiglylglycine emerge consistently across models.*

**Metabolic differences between controls and F1**

**Supplementary Figure S15:** *Metabolic differences between controls and F1 using OPLS-DA, RF, and SVM (observation diagnostics, OPLS-DA score plot, top contributing metabolites, and violin plots of lactic acid, citric acid, tiglylglycine, and β-hydroxybutyrate). Lactic acid, citric acid, and tiglylglycine emerge consistently across models.*

**Supplementary Figure S16:** *Machine-learning workflow for metabolomic classification: stratified train/test split; pre-processing (KNN imputation, centre/scale) fitted on training data only and applied to the test set; feature selection within training folds; model training (OPLS-DA, SVM, RF); cross-validation; independent test-set evaluation; and validation by ROC, permutation testing, and calibration.with hyperparameter tuning by inner cross-validation; and permutation-based validation.*
